# Cytokinin dehydrogenase: a genetic target for yield improvement in wheat

**DOI:** 10.1111/pbi.13305

**Published:** 2019-12-22

**Authors:** Lei Chen, Jiqiang Zhao, Jiancheng Song, Paula E. Jameson

**Affiliations:** ^1^ School of Life Sciences Yantai University Yantai China; ^2^ School of Biological Sciences University of Canterbury Christchurch New Zealand

**Keywords:** CKX, cytokinin oxidase/dehydrogenase, cytokinin, wheat, yield, TILLING, whole‐exome sequencing, root growth, mineral nutrition

## Abstract

The plant hormone group, the cytokinins, is implicated in both qualitative and quantitative components of yield. Cytokinins have opposing actions in shoot and root growth—actions shown to involve cytokinin dehydrogenase (CKX), the enzyme that inactivates cytokinin. We revise and provide unambiguous names for the *CKX* gene family members in wheat, based on the most recently released wheat genome database, IWGSC RefSeq v1.0 & *v2*.0. We review expression data of *CKX* gene family members in wheat, revealing tissue‐specific gene family member expression as well as sub‐genome‐specific expression. Manipulation of *CKX* in cereals shows clear impacts on yield, root growth and orientation, and Zn nutrition, but this also emphasizes the necessity to unlink promotive effects on grain yield from negative effects of cytokinin on root growth and uptake of mineral nutrients, particularly Zn and Fe. Wheat is the most widely grown cereal crop globally, yet is under‐research compared with rice and maize. We highlight gaps in our knowledge of the involvement of *CKX* for wheat. We also highlight the necessity for accurate analysis of endogenous cytokinins, acknowledging why this is challenging, and provide examples where inadequate analyses of endogenous cytokinins have led to unjustified conclusions. We acknowledge that the allohexaploid nature of bread wheat poses challenges in terms of uncovering useful mutations. However, we predict TILLING followed by whole‐exome sequencing will uncover informative mutations and we indicate the potential for stacking mutations within the three genomes to modify yield components. We model a wheat ideotype based on *CKX* manipulation.

## Introduction

Wheat is the most widely grown crop around the globe and ranks second in importance to rice for food (FAOSTAT, 2018). It is the second most important food crop in China (Ud Dowla *et al.*, 2018) and yet, in comparison with rice and maize, wheat is under‐explored (Schnurbusch, 2019). Clearly, there is a need to increase yield in the face of a world population increasing from the current 7.5 billion to 9 billion by 2050, and this is with the unprecedented challenges posed by climate change with its increased risk of drought (Hochholdinger, 2016; Hochman *et al.*, 2017; Ud Dowla *et al.*, 2018), concerns regarding the need to reduce N‐fertilizer use (Zörb *et al.*, 2018) and increasing disease risk (Gautam *et al.*, 2003; Zhang *et al.*, 2015). However, quality must also be considered, not only if the end product is for baking and processing (Zörb *et al.*, 2018) but also for when wheat is the staple food, consumed as the major source of carbohydrate and providing basic nutrition. Wheat can, for instance, be lacking in Zn and Fe, leading to micronutrient malnutrition (the ‘hidden hunger’) (Mayer *et al.*, 2018) in developing countries (see Beasley *et al.*, 2019; Ramireddy *et al.*, 2018a, b).

The Green Revolution cereals were developed at a time when the use of nitrogen fertilizer was increasing. The crops, selected for their semi‐dwarf, strong straw characteristics, yielded highly when supplied with N, water and treated with pesticides. Subsequently, it was shown that semi‐dwarf wheats and maize were gibberellin‐insensitive mutants, while the semi‐dwarf rice was a gibberellin biosynthetic mutant (Hedden, 2003). Significantly, these crops were not specifically selected for increased grain number or size—increased yield is considered due to reduced competition for assimilates from the shortened stem and reallocation of resources to the spikes increasing grain number (Fischer and Stockman, 1986).

The plant hormone group, the cytokinins, has been strongly implicated in many aspects affecting yield, particularly grain number and size (Jameson and Song, 2016; Yamburenko *et al.*, 2017), but including response to biotic and abiotic stressors (Cortleven *et al.*, 2019; Pavlů *et al.*, 2018), mineral status (Gao *et al.*, 2019; Guo *et al.*, 2017) and leaf senescence (Hönig *et al.*, 2018; Zwack *et al.*, 2013). The cytokinins, which are positive regulators of shoot growth and negative regulators of root growth (Werner *et al.*, 2003), are implicated in the control of both shoot architecture (Bartrina *et al.*, 2011; Han *et al.*, 2014) and root system architecture (Waidmann *et al.*, 2019; Werner and Schmülling, 2009), and crown root formation (Gao *et al.*, 2014). Recent research indicates a significant negative role in the control of micronutrient uptake (Gao *et al.*, 2019; Nehnevajova *et al.*, 2019; Ramireddy *et al.*, 2018a).

In this review, we emphasize aspects of cytokinin biology in cereals before focusing on the cytokinin catabolism gene family, cytokinin oxidase/dehydrogenase (CKX). We provide a brief introduction to the cytokinins and briefly introduce the components of yield in cereals and changes in endogenous cytokinins during grain development before revising the naming of the *CKX* gene families in wheat. As there are recent reviews covering the cytokinins in biotic and abiotic stress, and disease (Cortleven *et al.*, 2019), in nitrogen nutrition (Gu *et al.*, 2018) and senescence (Hönig *et al.*, 2018; Zwack and Rashotte, 2013), we focus on research implicating *CKX* in controlling grain yield in rice, barley and wheat, and in root growth and the uptake of micronutrients, and reveal gaps in our knowledge concerning wheat. Having identified these gaps, we foresee the use of mutants of specific *TaCKX* gene family members, identified through Targeting Induced Local Lesions IN Genomes (TILLING) followed by whole‐exome sequencing, in the creation of a wheat ideotype.

## Cytokinins

The committed step in the synthesis of the cytokinins occurs in two ways: either by an isopentenyl transferase (IPT) attaching an isoprenoid side chain to an ATP/ADP leading to the formation of the nucleotides of isopentenyl adenine (iP) and *trans*‐zeatin (tZ), or by a tRNA‐IPT leading, indirectly, to the *cisZ*‐type cytokinins. LONELY GUY (LOG) activates the cytokinin by releasing the free bases from the nucleotide forms, while destruction of cytokinin is carried out by cytokinin oxidase/dehydrogenase (CKX). Inactivation can also occur via glycosylation—either for storage by *O*‐glucosylation or for inactivation via N‐glucosylation. The active free base forms (*trans*‐zeatin (*t*Z), isopentenyl adenine (iP), dihydrozeatin (DHZ) and *cis*‐zeatin (*c*Z)) are detected by histidine kinase receptors, followed by a multistep phosphorelay to activate type A and type B response regulators (RRs). Type A RRs operate as a negative feedback system, whereas type B RRs are transcription factors that target primary cytokinin‐responsive genes. For more detail on cytokinin forms, function and signal transduction, see Jameson (2017), Kieber and Schaller (2018), Romanov *et al. *(2018) and Worthen *et al. *(2019).

It is critically important that analysis of cytokinin content is performed appropriately. There are over 20 different forms of cytokinins in wheat alone (Sýkorová *et al.*, 2008), most of which can interconvert to release the active free bases. Comprehensive analysis requires a variety of purification steps, separation, and identification and quantitation via MS/MS using appropriate internal standards (e.g. Dobrev and Vankova, 2012; Novák *et al.*, 2017; Powell *et al.*, 2013). It has never been acceptable to attempt to quantify cytokinins using UV absorbance following HPLC/UHPLC of partially purified extracts (e.g. as in Geng *et al.*, 2017; Nayar *et al.*, 2013), because the cytokinins are usually present in low ng to pg/g FW quantities, and UV‐absorbing impurities will mask the cytokinin. ELISA or RIA of partially purified but not chromatographically separated extracts is also unacceptable (see Sayavedra‐Soto *et al.*, 1988 for an example of the pre‐purification and separations steps required prior to immunoassay). Including an identification of kinetin, which has never been identified as naturally occurring, and adenosine (usually present at 1000 times greater quantity than the cytokinins) is indicative, not only of unacceptable technique, but also of a lack of understanding of the complexity of the cytokinins (e.g. as evidenced in Joshi *et al.*, 2018). Basunia and Nonhebel (2019) also emphasized this issue of inadequate analyses with regard to auxin and cytokinin analyses.

## Morphological components contributing to grain yield in wheat, barley and rice

In wheat and barley, the inflorescence is a spike, with the grain born within florets of the spikelets arranged on the spike. Yield is determined by 1000‐grain weight (TGW), the number of grains per spike and the number of spikes per area (i.e. tiller number) (Feng *et al.*, 2017). Tiller number is regulated genetically (Guo and Schnurbusch, 2015), but in the field, tiller number is determined to a greater or lesser extent by seed spacing at sowing. The spikelet of wheat is indeterminate and produces more than eight florets, whereas the inflorescence (i.e. the spike) is determinate (Feng *et al.*, 2017), and the spikelet number is set at the end of the double ridge stage (Guo and Schnurbusch, 2015; Ochagavía *et al.*, 2018). The terminal spikelet and floret differentiation have all occurred when the spike is around 2.0 mm in length (Gardner *et al.*, 1985). Most of the florets abort, leaving three to five grains at harvest (Feng *et al.*, 2017). Unlike wheat, barley has determinate spikelets (yielding two‐rowed or six‐rowed barley) but an indeterminate spike (refer Gauley and Boden, 2019).

In contrast to wheat and barley, the rice inflorescence is a panicle: yield is determined by TGW, the number of grains per panicle and the number of tillers (one panicle per tiller). Branching of panicles is set early and establishes the potential number of grains per tiller. Each spikelet bears one floret and, therefore, one grain (see Itoh *et al.*, 2005).

When collecting material for detailed analysis, it is important to recognize that, in wheat, anthesis starts in the upper middle of the spike and moves in a wave up and down the spike. Grains in the two basal florets of a spikelet are the largest. Poor filling occurs in higher‐level florets and in the last‐developed spikelets at the tip of the spike. Consequently, for clear developmental analyses, dissection of ovules/grains from the two basal florets from the middle section of a spike is important, as analyses of whole spikes will contain ovules/grains at varying developmental stages. The development of the barley spike follows a similar pattern to that in wheat. In rice, anthesis starts from the apical region of the panicle, and it takes about six days to reach the basal region. Poor grain filling occurs in basal spikelets.

## Endogenous changes in cytokinins during grain development

**Figure 1:**
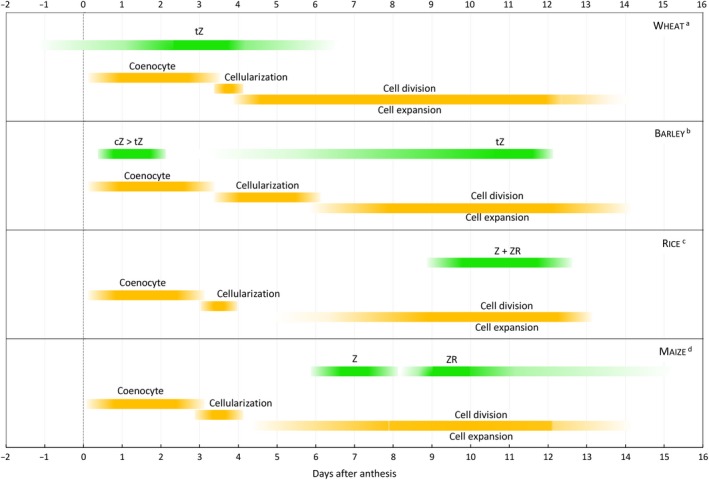
Generalized relationship between peak cytokinin and key changes during endosperm development. ^a^wheat (Banowetz et al., 1999; Gao et al., 1992; Hess et al., 2002; Jameson et al., 1982; Lenton and Appleford, 1986; Mares et al., 1977); ^b^barley (Powell et al., 2013; Sabelli and Larkins, 2009; Wilson et al., 2006; Zhang et al., 2016); ^c^rice (Brown et al., 1996; Chen et al., 2016; Folsom et al., 2014; Ishikawa et al., 2011); ^d^maize (Brugière et al., 2003, 2008; Hluska et al., 2016; Olsen, 2001; Rijavec et al., 2011).

The endosperm of cereals is the storage organ and a critical component of yield. Development is often divided into four stages: early development (double fertilization and syncytium formation); cellularization; differentiation (delineation of cell types, mitosis and endoreduplication, accumulation of storage materials); and finally, maturation (programmed cell death, dormancy and desiccation). All cereals, indeed all monocots and dicots, undergo a phase of free nuclear divisions resulting in a transitory coenocyte (Olsen, 2004). In wheat, barley, rice and maize, this is followed immediately by cellularization of the endosperm which is immediately followed by mitotic cell divisions, which have declined by about 12–14 days after anthesis (DAA) (Fig. 1). In cereals, cell number establishes final grain size. However, the transition from the syncytial stage to cellularization has been shown in rice to be critical. Precocious or delayed transition from the syncytium to cellularization in the endosperm is reported to cause abnormal seeds and, potentially, seed abortion in rice (Chen *et al.*, 2016; Folsom *et al.*, 2014).

The peak levels of cytokinin in barley, rice and maize are reported to occur around 10‐12 DAA (Fig. 1), although Hluska *et al. *(2016) suggest a much later cytokinin peak in maize kernels. It is not well recognized that the timing of changes in endogenous cytokinins in wheat differs from that in barley, rice and maize. In wheat, rapid changes in cytokinins occur around and immediately following anthesis, with a sharp peak of zeatin around 3‐4 DAA, at the onset of mitotic cell divisions (e.g. Hess *et al.*, 2002; Jameson *et al.*, 1982; Lenton and Appleford, 1986; Morris *et al.*, 1993) (Fig. 1).

However, there are over 20 different cytokinins present in wheat (Sýkorová *et al.*, 2008), but a comprehensive analysis over close time frames during spike, spikelet, ovule and grain development has not yet been reported for wheat (or rice) using LC‐MS/MS, nor have the unusual glycosides mentioned by Lenton and Appleford (1986) been identified. Most of the cytokinins have been ignored in recent papers, including the *O*‐glucosides. Cytokinins are present in the ovule around fertilization (Jameson *et al.*, 1982; Lenton and Appleford, 1986), but *trans*‐zeatin is the predominant form after anthesis (unpublished data). There is rapid metabolism of cytokinins during early wheat grain development, and this is matched by marked changes in gene expression. It is clear from the expression of *TaIPT, CKX, cZOG1* and ß‐glucosidase gene family members at 2 to 4 DAA that specific members of these four multigene families play key roles in determining the level of cytokinin during the phase of free nuclear and subsequent mitotic cell divisions (Song *et al.*, 2012).

## Phylogenetic analysis and renaming of *CKX* gene family members in bread wheat

Cytokinin oxidase/dehydrogenase has been depicted as a key enzyme regulating the cytokinin levels in cereals including maize (Brugière *et al.*, 2003), rice (Ashikari *et al.*, 2005), barley (Zalewski *et al.*, 2014) and wheat (Ogonowska *et al.*, 2019; Song *et al.*, 2012; Zhang *et al.*, 2012b). *CKX* belongs to a small gene family. *Arabidopsis thaliana* (arabidopsis) has seven homologues and rice 11, while in bread wheat 11 to 14 gene family members have been proposed (Mameaux *et al.*, 2012; Ogonowska *et al.*, 2019; Shoaib *et al.*, 2019; Song *et al.*, 2012). Recently, Ogonowska *et al. *(2019) and Shoaib *et al. *(2019) have suggested different numbering of the *TaCKX2.2, 3, 6, 9, 10* and *11* gene family members and allocation to chromosomes. Ogonowska *et al. *(2019) mainly followed the gene nomenclature suggested by former researchers (Lei *et al.*, 2008; Lu *et al.*, 2015; Mameaux *et al.*, 2012; Song *et al.*, 2012; Zhang *et al.*, 2012b), and allocated the *TaCKX2* family to two subfamilies *TaCKX2.1* and *TaCKX2.2* (Table 1). Shoaib *et al. *(2019) renamed most of the *TaCKX* gene family members based on a comprehensive phylogenetic analysis. In detail, *TaCKX3*, *TaCKX6*, *TaCKX9*, *TaCKX10* and *TaCKX11* that had been named by former researchers were renamed *TaCKX11*, *TaCKX3*, *TaCKX10*, *TaCKX9* and *TaCKX8*, respectively (Table 1; Fig. S1). In addition, Shoaib *et al. *(2019) suggested that five genes, that is TraesCS3A01G311100, TraesCS3B01G161000, TraesCS3D01G143500, TraesCS3D01G143300 and TraesCS3D01G143200, be given new subfamily names *TaCKX14A*, *TaCKX14B*, *TaCKX12D*, *TaCKX14D* and *TaCKX13D*, respectively (Table 1). Notably, all of these five genes were classified as the *TaCKX2.2* subfamily by Ogonowska *et al. *(2019).

**Table 1:** Revised naming of the *CKX* gene family members in wheat

Current suggestion	Gene ID (RefSeq v1.0)	Ogonowska *et al. *(2019)	Shoaib *et al. *(2019)	Song *et al. *(2012)	Others (from Oganowska *et al.*, 2019)
*TaCKX1‐3A* *TaCKX1‐3B* *TaCKX1‐3D*	TraesCS3A01G109500 TraesCS3B01G128700 TraesCS3D01G111300	*TaCKX1*	*TaCKX1A* *TaCKX1B* *TaCKX1D*	*TaCKX1*	
*TaCKX2.1‐3A* *TaCKX2.1‐3B* *TaCKX2.1‐3D* *TaCKX2.2.1‐3A* *TaCKX2.2.1‐3B* *TaCKX2.2.1‐3D* *TaCKX2.2.2‐3D* *TaCKX2.2.3‐3D*	TraesCS3A01G311000 TraesCS3B01G161100 TraesCS3D01G143600 TraesCS3A01G311100 TraesCS3B01G161000 TraesCS3D01G143500 TraesCS3D01G143300 TraesCS3D01G143200	*TaCKX2.1*	*TaCKX2A* *TaCKX2B* *TaCKX2D* *TaCKX14A* *TaCKX14B* *TaCKX12D* *TaCKX14D* *TaCKX13D*	*TaCKX2*	HM195292 *TaCKX2.5* *TaCKX2.3* *TaCKX6a02* *TaCKX2.4* *TaCKX2.1* *TaCKX6D1* *TaCKX2.2* HM195293
		*TaCKX2.2*			
*TaCKX3‐1A* *TaCKX3‐1B* *TaCKX3‐1D*	TraesCS1A01G159600 TraesCS1B01G176000 TraesCS1D01G157000	*TaCKX6*	*TaCKX3A* *TaCKX3B* *TaCKX3D*	*TaCKX6*	
*TaCKX4‐3A* *TaCKX4‐3B* *TaCKX4‐3D*	TraesCS3A01G481000 TraesCS3B01G525300 TraesCS3D01G475800	*TaCKX4*	*TaCKX4A* *TaCKX4B* *TaCKX4D*	*TaCKX4*	
*TaCKX5‐3A* *TaCKX5‐3B* *TaCKX5‐3D*	TraesCS3A01G321100 TraesCS3B01G344600 TraesCS3D01G310200	*TaCKX5*	*TaCKX5A* *TaCKX5B* *TaCKX5D*		
*TaCKX7‐6A* *TaCKX7‐6B* *TaCKX7‐6D*	TraesCS6A01G185800 TraesCS6B01G214700 TraesCS6D02G172900	*TaCKX7*	*TaCKX7A* *TaCKX7B* *Wrong ID*	*TaCKX7/8*	
*TaCKX8‐2A* *TaCKX8‐2B* *TaCKX8‐2D*	TraesCS2A01G378300 TraesCS2B01G395200 TraesCS2D01G374600	*TaCKX11*	*TaCKX8A* *TaCKX8B* *TaCKX8D*	*TaCKX11*	
*TaCKX9‐1A* *TaCKX9‐1B* *TaCKX9‐1D*	TraesCS1A01G234800 TraesCS1B01G248700 TraesCS1D01G237200	*TaCKX10*	*TaCKX9A* *TaCKX9B* *TaCKX9D*	*TaCKX10*	
*TaCKX10‐7A* *TaCKX10‐7B* *TaCKX10‐7D*	TraesCS7A01G363400 TraesCS7B01G264400 TraesCS7D01G359700	*TaCKX9*	*TaCKX10A* *TaCKX10B* *TaCKX10D*	*TaCKX9*	
*TaCKX11‐7A* *TaCKX11‐7B* *TaCKX11‐7D*	TraesCS7A01G536900 TraesCS7B01G455000 TraesCSUn01G106300	*TaCKX3*	*TaCKX11A* *TaCKX11B* *TaCKX11D*	*TaCKX3*	

To avoid further confusion, we mined the most recent wheat databases including the IWGSC RefSeq v1.0 and the corresponding genome feature format annotation file (gff3 file), as well as the just released, updated genome sequence IWGSC RefSeq v2.0 (no gff3 file). By comparing the recent numbering of *CKX* homologues in closely related monocot species including rice, maize, barley, *Aegilops tauschii*, *Setaria italica* and *Brachypodium distachyon*, we suggest renumbering the family members as *TaCKX1, TaCKX2.1, TaCKX2.2.1, TaCKX2.2.2, TaCKX2.2.3, TaCKX3, TaCKX4, TaCKX5, TaCKX7, TaCKX8, TaCKX9, TaCKX10* and *TaCKX11* (Table 1; Fig. S1).

**Figure 2:**
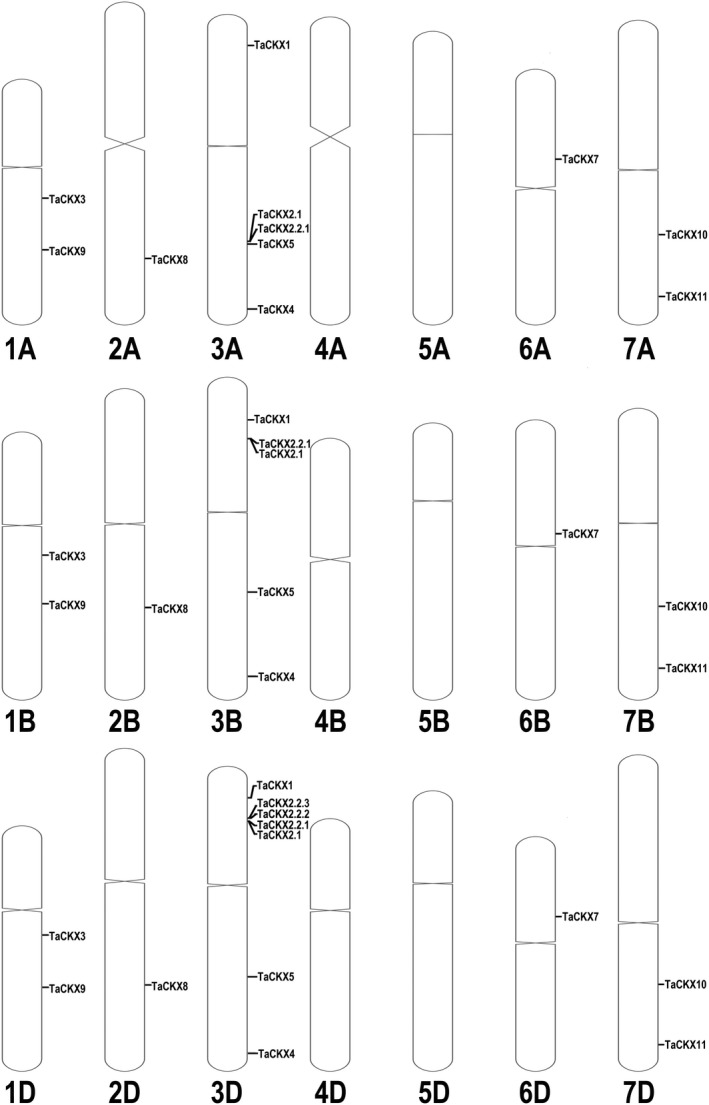
Positions of *TaCKX* gene family members on each of the A, B and D wheat chromosomes. The chromosome length and centromere data sets were obtained from IWGSC (Science 361, 2018). Gene positions, except for *TaCKX11‐7D*, were extracted from IWGSC RefSeq v1.0 gff3 file using the awk program in the linux system. The position of *TaCKX11‐7D* was determined by local blast in the IWGSC RefSeq v2.0 genome sequence. All of the *TaCKX* genes were mapped to their corresponding chromosome using the R package RIdeogram (v0.1.1). Group maps were merged and edited in Photoshop CS6.

Additionally, we mapped all of the *TaCKX* family genes onto their corresponding positions on each chromosome (Fig. 2). Notably, one of the *TaCKX11* subfamily, *TaCKX11‐ChrUn*, had not been mapped to a position on any one of the 21 chromosomes in the IWGSC RefSeq v1.0 genome sequence. However, we mapped it onto the 7D chromosome position 622518348–622521325 in our local BLAST database, which was developed using the IWGSC RefSeq v2.0 genome sequence. Thus, all 35 *TaCKX* gene family members can now be allocated to one of the three chromosomes associated with the A, B and D genomes (Fig. 2). Finally, to indicate on which chromosome the gene family member is located, the renumbered gene names have also been marked by their allocated chromosome number (Table 1; Fig. S1).

**Figure 3:**
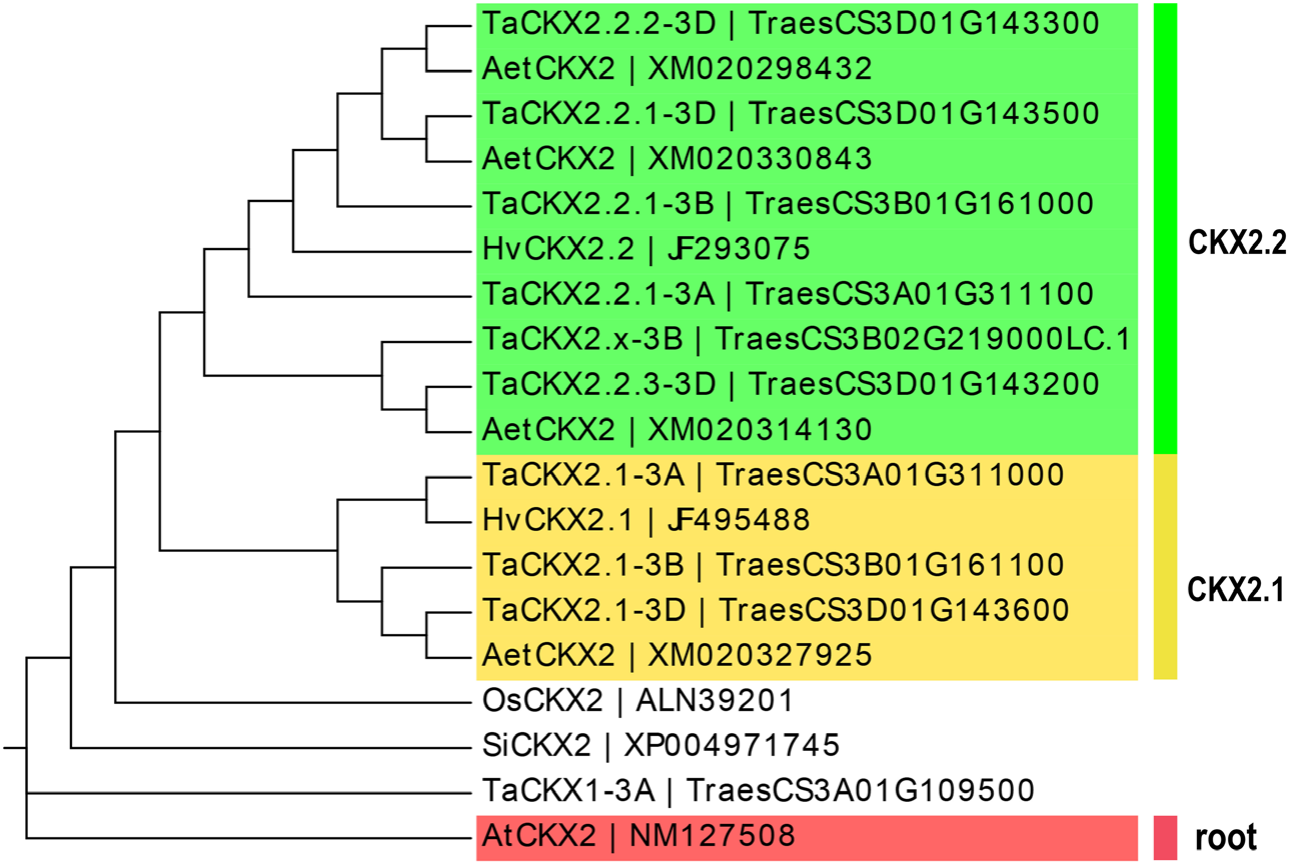
Phylogenetic cladogram showing the separation of the *TaCKX2.1* and *TaCKX2.2* paralogues into two sub‐gene families. Neighbour‐joining phylogenetic tree generated based on protein sequences in *Triticum aestivum* (Ta), *Hordeum vulgare* (Hv), *Oryza sativa* (Os)*, Zea mays* (Zm), *Aegilops tauschii* (Aet), *Setaria italica* (Si) and *Arabidopsis thaliana* (At). The tree was rooted using *CKX2* sequence of *Arabidopsis thaliana* (At). Node values are percentages of bootstraps generated with 1000 bootstrap replicates. The cladogram was drawn using the online tool Evolview v1 and v2 (Zhang *et al*., 2012a; He *et al*., 2016).

Additionally, *TaCKX2* has undergone gene duplication (Mameaux *et al.*, 2012), with Lu *et al. *(2015) suggesting that the *TaCKX2s* on chromosome 3D could be subdivided into two groups based on their homology, as shown by Ogonowska *et al. *(2019). The phylogenetic tree shown in Figure 3 separates the paralogues into two sub‐gene families (*CKX2.1* and *2.2*). Notably, *HvCKX2.1* and *HvCKX2.2* from *Hordeum vulgare* grouped with the *TaCKX2.1* and *TaCKX2.2* sub‐clusters, respectively. However, the *TaCKX2.2* sub‐gene family has expanded and has more members compared with *HvCKX2.2*. Functional investigation of these new members is critical as they could be of considerable importance for genetic improvement.

After locating all the duplicated paralogues on their respective chromosomes, we also noted that the location of *TaCKX2.1* and *TaCKX2.2.1* on the 3A long arm contrasts to their location on the short arm of 3B and 3D (Fig. 2). We suggest that this may be a non‐reserved transposition event from the 3A short arm to the 3A long arm, and this also resulted in the reversal of the position of *TaCKX2.1* relative to *TaCKX2.2.1* compared with that of 3B and 3D (Fig. 2). Similarly, we suggest that the *TaCKX2.2.2* and *TaCKX2.2.3* paralogues located on the 3D chromosome may also be a result of another transposition event, in this case a replicative transposition which produced two paralogues.

We suggest the following naming for these paralogues: *TaCKX2.1‐3A* and *TaCKX2.2.1‐3A,* and *TaCKX2.1‐3B* and *TaCKX2.2.1‐3B* on chromosomes 3A and 3B, respectively, and four *TaCKX2* paralogues, *TaCKX2.1‐3D*, *TaCKX2.2.1‐3D, TaCKX2.2.2‐3D* and *TaCKX2.2.3‐3D* on chromosome 3D (Table 1). Shoaib *et al. *(2019) named these paralogues individually as 2, 12, 13 and 14, whereas Ogonowska *et al. *(2019) had allocated these paralogues to two distinct gene families—*CKX2.1* and *CKX2.2.*

**Figure 4:**
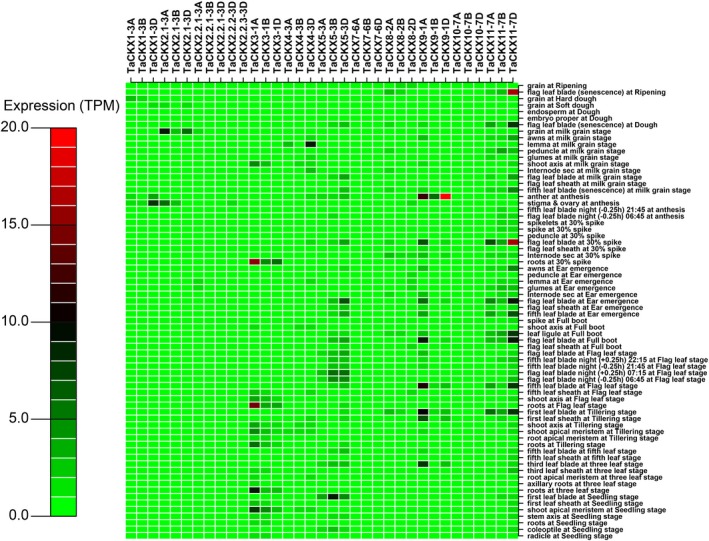
Heat map depicting expression of *TaCKX* gene family members based on RNA‐seq data. The raw data sets were collected from IWGSC (Science 361, 2018) and calculated by Wheat Omics of China. We extracted the *TaCKX* gene expression data set using awk, python and perl programs. The figure was drawn using the expression mean value in R language.

Individual *CKX* gene family members are expressed in different tissues (Werner *et al.*, 2006). For wheat, this has been shown through gene expression analyses (Ogonowska *et al.*, 2019; Song *et al.*, 2012) and can be found in the comprehensive RNA‐seq data set from the IWGSC (2018), where expression data were obtained from hundreds of RNA‐seq samples. By abstracting the *TaCKX* gene family members from data on roots, leaves, spike and grain, tissue specificity in wheat is clear, as is differential expression between sub‐genomes (Fig. 4; Fig. S2). *TaCKX1* is most expressed during grain development, with sub‐genome D more highly expressed in the stigma + ovary at anthesis, sub‐genome B in the grain at the milk stage and sub‐genome A at the soft dough stage (Fig. 4 and Fig. S2A). *TaCKX2* gene family members also expressed during grain development, with *TaCKX2.1* more highly expressed than the *TaCKX2*.2 sub‐gene family (Fig. S2B–F). Combining all eight *TaCKX2* gene family members, it is clear that expression is targeted to the developing grain, with somewhat greater expression at the milk grain stage than in the ovary + stigma at anthesis (Fig. S2F).

*TaCKX3* was one of the more highly expressed gene family members with expression restricted to vegetative tissues: the roots at the tillering, flag leaf and the 30% spike stages, shoot apical meristem (SAM) at the seedling and tillering stages, and the shoot axis at the milk grain and full boot stages (Fig. 4; Fig. S2G). *TaCKX4‐3D* did not express in the grain either, but in the lemma at the milk stage and at low levels in roots (Fig. 4; Fig. S2H). *TaCKX5‐3B* and *TaCKX5‐3D* expressed in most leaf blades including the flag leaf blade, with only low expression in the ovary at anthesis (Fig. 4; Fig .S2I).

*TaCKX8* expressed mostly in the first leaf blade at tillering, the internode at 30% spike and the senescing flag leaf (Fig. 4; Fig. S2K). *TaCKX11*, and particularly *TaCKX11‐7D*, had low expression at early stages of development, but expressed in leaves at the tillering stage and in the flag leaf blade at full boot, ear emergence and 30% spike stage. *TaCKX11*‐*7D* was the most highly expressed *TaCKX* gene family member during leaf senescence (Fig. 4; Fig. S2N). *TaCKX9* expressed strongly in anthers at anthesis, while *TaCKX9‐1A* expressed in leaf blades and in the flag leaf (Fig. 4; Fig. S2L).

Both *TaCKX7* and *10* were expressed at very low levels, *TaCKX10* principally during early root growth (Fig. S2M), whereas *TaCKX7‐6B* expressed later in roots at the flag leaf and 30% spike stages (Fig. S2J). Overall, these data are broadly similar to the RT‐qPCR data published by Ogonowska *et al. *(2019). However, an important time window of seed development, between anthesis and milk stage, was not included in the RNA‐seq data set: *TaCKX1* and *TaCKX2* gene family members are particularly highly expressed at 1‐14 DAA (Ogonowska *et al.*, 2019; Song *et al.*, 2012; Zhang *et al.*, 2012b).

## Associations between CKX and seed yield components

Bread wheat is a recent polyploid species, so there has been a relatively short time for diploidization—the process that dilutes and gradually erases the previous duplication (Uauy *et al.*, 2017). Consequently, most genes are expected to have overlapping functions, although divergence amongst the three diploid genomes since their last common ancestor is to be expected (Uauy *et al.*, 2017). Further, these authors suggest the effects of gene mutations in recent polyploid species will more frequently be masked by the other genomes. However, if a unique allele is identified in a landrace or modern variety but is absent from the wild progenitors, it is likely to have been selected during domestication. Both forward and reverse genetic approaches have been utilized that show that *CKX* gene family members are implicated in yield in rice, barley and wheat.

### CKX and yield in rice

There are a number of publications linking poor grain filling in rice to lower cytokinin content, and particularly to greater levels of *OsCKX* expression/ enzyme activity in different cultivars or in apical versus basal spikelets (see Panda *et al.*, 2018 and references therein).

Since Ashikari’s seminal work with rice (Ashikari *et al.*, 2005), CKX has been identified as having a key role in yield determination (summarized in Table 2). Ashikari *et al. *(2005) showed that the rice *Gn1a* QTL on chromosome 1 linked to increased yield is a gene for *CKX2*. Overexpressing or reducing activity of *Gn1a* reduced or increased grain number, respectively. A null mutation of *OsCKX2* increased grain number in line 5150, a high yielding Chinese variety. When expression of all 11 rice *CKX* genes was compared, *OsCKX2* showed greatest expression in the cv Koshishikari (which had the lowest grain number) and none of the other 10 *CKXs* was differentially expressed amongst the lines assessed pointing to *OsCKX2* having a preeminent role in controlling yield.

**Table 2:** Impacts on yield components of *CKX* manipulations

Plant	*CKX* family member	Alteration	Effect	References
Rice	*CKX2* [Gn1a]	*ckx2* mutant	↑ panicle branches ↑ grains/panicle ↑ grain number	Ashikari *et al. *(2005)
		RNAi	↑ grain number	
		overexpression	↓ grain number	
	*CKX2*	RNAi	↑ tiller number ↑ grain number ↑ grain weight ns grains/panicle	Yeh *et al. *(2015)
	*CKX2* [Gn1a]	CRISPR/Cas9	↑ panicle size ↑ flower number [yield not reported]	Li *et al. *(2016)
	*CKX9* [responds only to strigolactone]	CRISPR/Cas9 overexpression	↑ tiller number ↓ panicle size ↓ grain number	Duan *et al. *(2019)
	*CKX2* papers		Inadequate/misleading cytokinin measurements	Joshi *et al. *(2018), Nayar *et al. *(2013)
	*LP*	mutant *lp*	↑ panicle size ↑ grain number ↓ *OsCKX2*	Li *et al. *(2011)
Barley	*HvCKX1*	RNAi	↑ spike number in T4 ↑ grain number in T4	Zalewski *et al. *(2014)
	*HvCKX9*	RNAi	no effect at T4	
	*HvCKX1*	RNAi	↑ spike number	Holubová *et al. *(2018)
			↑ grain number	
			↓ 1000 grain weight	
			↑ yield	
		CRISPR/Cas9	no yield data provided	
	*HvCKX1*	CRISPR/Cas9	limited effect	Gasparis *et al. *(2019)
	*HvCKX3*	CRISPR/Cas9	↓ grain number	
			↓ grain weight	
Wheat	*Ta2.2.1‐3A*	RNAi	↑ grain number/spike No change in spikelet number	Li *et al. *(2018)
	*GW2*	CRISPR/Cas9 plus TILLING; triple mutant on A, B, D sub‐genomes	↑ grain size ↑ 1000 grain weight	Wang *et al. *(2018)
	*GW2* paper	Link between GW2 and cytokinin not validated	Inadequate cytokinin measurements	Geng *et al. *(2017)
	*CKX2.2.1‐3D*	Mutation association analysis	↑ 1000 grain weight	Zhang *et al.* (2012b)
	*CKX2.1‐3D*	Mutation association analysis	↑ grain size ↑ grain weight ↑ grain filling rate	Lu *et al. *(2015)
	*CKX4‐3A, 3D*	Variant association analysis	↑ grain weight	Chang *et al. *(2015)

More recently, Li *et al. *(2016) used gene editing to target mutations to *Gn1a* of rice cultivar Zhonghua 11, a widely grown modern *japonica* cultivar in China. They found by editing in a mutation that led to a frameshift in *OsCKX2*, plant height increased, as did panicle size and number of flowers per panicle, but they did not report on grain number or 1000‐grain weight (Table 2).

Investigating the association between grain number and grain size, Guo *et al. *(2018) suggest that *GRAIN SIZE AND NUMBER (GSN1)* controls the trade‐off between grain size and number in rice, at least partly through *CKX2*. They showed that *CKX2* was up‐regulated in the *gsn1* mutant and cytokinin levels reduced during young panicle development, with a consequent increase in grain size but a less branched panicle and overall fewer grains than the wild type. This supports a role for CKX2/cytokinin in the control of panicle architecture and, consequently, grain number in rice (Ashikari *et al.*, 2005; Li *et al.*, 2016).

Mutations in the rice F‐box gene, *LARGER PANICLE* (*LP*, a component of the ubiquitin‐mediated pathway), enhanced the yield of rice (Li *et al.*, 2011), through changes in panicle size and increased grain number. The plants were slightly taller and more resistant to lodging, with stronger culms and more vascular bundles. *OsLP* was shown to express in several tissues with *in situ* hybridization locating expression in primary and secondary branch primordia. Expression analysis in two allelic mutants revealed strong down‐regulation of *OsCKX2.* Li *et al. *(2011) concluded that LP might be involved in moderating the cytokinin level through direct or indirect control of the *OsCKX2* expression. However, a route between the reduced expression of *CKX* and the ubiquitin–proteasome system has yet to be shown.

An interesting interaction between strigolactones (SL) and cytokinins in rice was recently revealed, in which SL‐regulated tiller development was shown to operate through transcriptional activation of *OsCKX9* (Duan *et al.*, 2019). *OsCKX9* expressed in all tissues at the heading stage with greatest expression at the shoot bases. Both a gene‐edited mutant, *Osckx9,* and overexpression of *OsCKX9* increased tiller number, reduced plant height and decreased panicle size and grain number (Duan *et al.*, 2019). Strigolactones are a group of carotenoid‐derived plant hormones known to inhibit branching (Dun *et al.*, 2012), while a SL signalling mutant of rice has increased tillering and panicle branching. Notably, *OsCKX9* was shown to be unresponsive to cytokinin but to respond specifically to SL. Duan *et al. *(2019) showed that SL acts through elevation of *OsCKX9* expression, decreasing both cytokinin level and *OsRR5* expression. Earlier, Tsai *et al. *(2012) had shown expression of *OsCKX9* to be shoot‐specific, and while showing other *OsCKXs* to be responsive to cytokinin, they did not show data for *OsCKX9*.

However, it is important to recognize that increased tiller number is not likely to be beneficial to yield. Indeed, the aim of the new plant‐type rice ideotype was to have low tillering capacity and no unproductive tillers (Rubia *et al.*, 2014).The recently published paper by Zhao *et al. *(2019) implicated wheat TaD27‐B (an orthologue of arabidopsis and rice D27, a SL biosynthetic enzyme) in mediating tiller number in wheat through modifying the size of the axillary buds. These authors suggest yield in wheat can be increased by manipulating SL biosynthesis (decreasing it) to increase tiller number. Indeed, a barley line carrying a mutation in strigolactone signalling (*hvd14.d*) was previously shown to produce a greater number of tillers (Marzec *et al.*, 2016). However, multiple small tillers are considered undesirable as they redirect resources away from the main tillers and ultimately decrease yield (Hendriks *et al.*, 2016; Kebrom *et al.*, 2012).

While the focus has been on *OsCKX2* for obvious reasons, the effect of targeting the more highly expressed *OsCKX* family members such as *OsCKX11,* which is the most highly expressed gene family member in the early rice panicles (Yamburenko *et al.*, 2017), is yet to be investigated. These authors also point out that there is greater variation amongst the genes for cytokinin metabolism and type A RRs, than for genes in the primary signalling pathways, so that regulation of cytokinin levels is more through metabolism and negative feedback from the type A RRs, than through perception and the signal transduction pathway. Supporting this contention is the recognition that mutants of *OsLOG* (LOG converts cytokinin nucleotides to active free bases) have smaller panicles (Kurakawa *et al.*, 2007). However, the obvious necessity for a functional signal transduction pathway was recently shown in rice (Worthen *et al.*, 2019).

As mentioned earlier, cytokinin analyses must be unambiguous. Two papers on *CKX2* in rice give cause for concern. Joshi *et al. *(2018) focused on salt tolerance, and Nayar *et al. *(2013) focused on *MADS29*. The techniques used by both Joshi *et al. *(2018) and Nayar *et al. *(2013) were inadequate and neither article should be taken as supporting information for a role for CKX and/or cytokinins in elevating yield under salinity stress (Joshi *et al.*, 2018), or for the cytokinin pathway being a major target for *MADS29* (Nayar *et al.*, 2013). Careful work is needed to re‐evaluate these claims, especially in the light of several articles indicating that cytokinin‐*deficient* plants are more tolerant of salt stress than wild type (see Cortleven *et al.*, 2019).

### CKX and yield in barley

Following RNAi targeting of *HvCKX1* and *HvCKX9* by Zalewski *et al. *(2010, 2012), selected because they both express in developing kernels (Zalewski *et al.*, 2014), Holubová *et al. *(2018) used both hairpin RNAi targeted to ‘knockdown’ (KD) *HvCKX1* and a CRISPR/Cas construct targeted to the first exon of *HvCKX1* to ‘knockout’ (KO) *CKX1*. Reduction in *HvCKX1* expression and CKX activity was shown in selected RNAi KD lines. No effect was shown on the expression of other *HvCKX* or *HvIPT* genes indicating that the targeting was specific to *CKX1*. CKX activity was significantly decreased in the KO lines at all stages measured.

The cytokinin content of the KD and KO lines was carefully assessed: increases in *cis- * and *trans*‐zeatin (Z)‐type cytokinins and in isopentenyl (iP)‐type cytokinins were detected (Holubová *et al.*, 2018). Generally, the *c*Z types decreased with development, while the *t*Z types increased. Overall, the KD or KO of *HvCKX1* led to an increase in total cytokinin content, although there were differences, as might be expected, between individual cytokinin types and lines, in field and glasshouse‐grown plants (Holubová *et al.*, 2018).

Although variable in terms of growth stage and line, decreased root growth was generally seen in both KD and KO plants grown in hydroponics (Holubová *et al.*, 2018). In terms of yield, of the KD lines grown in the glasshouse, two showed increased spike numbers and one line substantially increased grain number leading to an overall increase in yield even though the TGW was reduced. Similarly, for field‐grown KD lines: spike numbers were increased, TGW was decreased, and an increase in yield was shown for one line over two seasons. No yield data were provided for the KO lines. These data from Holubová *et al. *(2018) are in agreement with the T4 generation of RNAi‐*CKX1* silenced barley plants, where increased yield was influenced by increased grain number, to a lesser extent by increased spike number, even though TGW was reduced (Zalewski *et al.*, 2014).

In the most recent barley paper, Gasparis *et al. *(2019) generated gene‐edited mutants of *HvCKX1* and *HvCKX3,* both of which are reported to be expressed in developing spikes of WT 0–14 days after pollination (DAP), and in seedling roots. In the Hvckx1 lines, a significant decrease in CKX activity was measured in 7 DAP spikes and in the seedling roots. In contrast, in the Hvckx3 lines, CKX activity in the spike was little changed relative to control, but a significant increase in CKX activity was observed in the roots, as was a significant decrease in *HvIPT10* expression. There was no consistency between lines in terms of yield components: notably, there was limited effect for ckx1 mutants; and apart from one ckx3 line showing an increased number of spikes, other lines showed decreased numbers of grains and grain weight. Based on gene expression combined with RNA‐seq data, the authors explain the limited effect on yield by the activation of strong homeostatic mechanisms via reduced *IPT* expression and enhanced inactivation via *0*‐glucosylation. They suggest yield increases noted in RNAi‐CKX plants (Zalewski *et al.*, 2010) may have occurred because the lesser reduction in CKX may not have invoked the strong up‐regulation of homeostatic mechanisms as seen with the knockout gene‐edited plants (Gasparis *et al.*, 2019). This observation may be pertinent to the gene‐edited Osckx9 mentioned earlier*,* where the morphology was similar to lines overexpressing *CKX9.* Duan *et al. *(2019) offered no explanation for their apparently contradictory results.

Interestingly, the root phenotypes of T_2_ plants showed opposite effects for the ckx1 and ckx3 mutants: ckx1 mutants generally showed enhanced root parameters, whereas ckx3 mutants showed inhibited root growth relative to control. This was potentially due to enhanced CKX9 activity and up‐regulation of type A RRs in the ckx1 mutant. The unexpected, negative effect of the ckx3 mutant is explained by perturbations in cytokinin signalling via up‐regulation of the AHP4 component of the signal transduction pathway (Gasparis *et al.*, 2019). Evidently, the homeostatic control mechanisms operate just as strongly in roots as they do in the shoot.

## CKX and yield in wheat

### TaCKX alleles associated with yield in wheat

Based on the hypothesis that, as in rice, wheat *CKX* genes might regulate agronomic traits and be useful to improve productivity, Zhang *et al. *(2012b) adopted a candidate gene approach. They focused on finding the orthologue of *OsCKX2*. They showed *TaCKX6* (renamed *TaCKX2.2.1‐3D*) to be the wheat orthologue of rice *OsCKX2* and to be located on chromosome D. To determine the function of *TaCKX2.2.1‐3D* in wheat, they undertook linkage analysis, association analysis and gene expression. Cultivar Yanzhan, which has an 18‐bp deletion in intron 2 of *CKX2.2.1‐3D*, was shown to be associated with a greater TGW than Neixiang 188. They confirmed this by testing for association between the 18‐bp deletion and TGW across 115 wheat accessions and concluded that *TaCKX2.2.1‐3D* may underlie the greater or lesser TGW of individual cultivars.

Expression analysis showed transcripts of *TaCKX2.2.1‐3D* reaching a maximum level in grains 8 DAP (Zhang *et al.*, 2012b), in a similar pattern to that shown by Song *et al. *(2012) for *TaCKX2* (renamed 2.2‐1) in winter bread wheat variety Equinox, and in the RNA‐seq data set (Fig S2C,F). A comparison of *TaCKX* expression between haplotypes carrying the deletion (haplotype *a*) and those without the deletion (haplotype *b*) showed significantly reduced *CKX* expression in those with haplotype *a*. The deletion was associated with increased TGW (Zhang *et al.*, 2012b).

Increased grain size is associated with domestication of cereals (Purugganan and Fuller, 2009). Zhang *et al. *(2012b) further showed that the karyotypes carrying the deletion in *TaCKX2.2.1‐3D* are essentially restricted to landraces and modern cultivars indicating that the deletion associated with increased TGW is likely to have been derived relatively recently. Because of the domestication bottleneck, the *TaCKX6*‐*D1* locus has little remaining genetic variation in either Chinese landraces or cultivated varieties (Zhang *et al.*, 2012b).

Zhang *et al. *(2012b) also suggest that functional divergence has occurred in CKX2, as *OsCKX2* is associated with grain number (Ashikari *et al.*, 2005), and *TaCKX2.2.1‐3D* is associated with grain weight. Expression analyses across various organs confirm this suggestion, with *TaCKX2.2.1‐3D* expressing mainly in kernels and relatively less in inflorescences (Zhang *et al.*, 2012b), whereas *OsCKX2* is mainly expressed in inflorescences and flowers (Ashikari *et al.*, 2005).

Lu *et al. *(2015) also isolated a novel allele of *CKX2*. *TaCKX6a02* has been allocated to the *TaCKX2*.*1* gene family (Ogonowska *et al.*, 2019) and renamed *TaCKX2.1‐3D* (Table 1). Lu *et al. *(2015) showed this gene was located to chromosome 3D. They set out to identify allelic variations of *CKX* genes for grain filling, grain size and grain weight using 169 recombinant inbred lines (RILs) developed by a cross between a high yielding winter wheat variety and a low yielding Chinese landrace differing in grain size, grain weight and grain filling, along with 102 other wheat varieties. Of the six *TaCKX* gene families assessed, only *TaCKX2*.*1‐3D* had a significant correlation with grain size, weight and grain filling rate. Two alleles were identified, with one, *TaCKX2.1‐3Da*, having significantly higher values for these characteristics than varieties with allele ‘*b’*. Following an association analysis amongst the 102 cultivars, 68% had the ‘*a’* allele, while the rest had the ‘*b’* allele. They concluded through the analysis of different genetic backgrounds, RILs and natural populations that the ‘*a*’ allele was positively correlated with improved yield characteristics. A 29‐bp insertion in the 3′ UTR region after the stop codon was detected in this allele compared with allele ‘*b*’. A full‐length sequence of this gene still needs to be obtained, and an expression analysis of *TaCKX2.1‐3D* is still to be carried out, as is a comparative analysis of *CKX* expression between lines carrying the two different alleles. However, the RNA‐seq database analysis indicates that peak expression of *TaCKX2.1‐3D* is in grains at the milk stage (Fig S2B), a stage relevant to grain filling and ultimate grain weight.

Chang *et al. *(2015), using the same RILs and varieties as described above for Lu *et al. *(2015), searched for alleles significantly associated with chlorophyll content and grain weight. Allelic variation between the two parental lines showed genotype A to contain two alleles, *TaCKX4‐1* and *TaCKX4‐2* (corresponding to *TaCKX4‐3A* and *TaCKX4‐3D* in Fig. S1) that were absent in genotype B. Only genotype A was positively associated with chlorophyll content and grain yield. However, no expression analysis has been carried out, nor full‐length sequences obtained. While the RNA‐seq data set (IWGSC, 2018) indicates *TaCKX4‐3D* is strongly expressed in the lemma at the milk stage and is also expressed in the shoot axis (Fig. 4; Fig. S2H), more information is needed concerning the nature of the *CKX* variations in the RILs.

### Down‐regulated CKX genes and yield in wheat

Grain number per spike in wheat was enhanced when a conserved fragment of *TaCKX2.2.1‐3A* (originally *TaCKX2.4*) was targeted by RNA interference (Li *et al.*, 2018). Li *et al. *(2018) showed a strong correlation between grain number and reduced expression of *TaCKX2.2.1‐3A* in T_3_ plants. Moreover, increased grain number was due to an increased grain number per spike. As there was no increase in spikelet number, this implies more filled florets. In the light of the results for rice where *OsCKX2* was associated with grain number (Ashikari *et al.*, 2005), and wheat where *TaCKX2.2.1‐3D* was associated with grain weight (Zhang *et al.*, 2012b), the results by Li *et al. *(2018) suggest a difference in function between the *TaCKX2.2.1* genes on chromosomes 3A and 3D. This leads to the potential of stacking mutations in *TaCKX2.2.1‐3D* and *TaCKX2.2.1‐3A* to address both seed number and seed size in bread wheat, even though the RNA‐seq data show similar profiles for both gene family members (Fig S2C).

GW2 has been associated with grain weight in rice and bread wheat, particularly wider grains and greater TGW (Geng *et al.*, 2017; Hong *et al.*, 2014; Simmonds *et al.*, 2016; Song *et al.*, 2007; Su *et al.*, 2011; Wang *et al.*, 2018), and there are suggestions that this is via cytokinin (Geng *et al.*, 2017; Sestili *et al.*, 2019). However, in both these cases the analyses need to be repeated using LC‐MS/MS for cytokinin analysis and appropriate reference genes for gene expression (Geng *et al.*, 2017), and to include the study of gene expression at earlier stages of development (Sestili *et al.*, 2019) to substantiate any link between GW2 and cytokinin.

## CKX is associated with root growth and mineral enrichment in cereals

It is well recognized that cytokinin levels in roots are supra‐optimal: overexpression of *AtCKX* leads to enhanced root growth, including greater lateral root density (Werner *et al.*, 2003; Werner and Schmülling, 2009), while elevated cytokinin inhibits root growth (McKenzie *et al.*, 1998; Zou *et al.*, 2018). However, cytokinin has been shown to be required for maintenance of both the shoot apical meristem and root apical meristem (Werner *et al.*, 2003; Wybouw and De Rybel, 2019). Additionally, cytokinin is involved in regulating root system architecture in arabidopsis: it acts as a positional cue through its interaction with auxin, regulating lateral root spacing through the action of *IPT* and *LOG4* genes (Chang *et al.*, 2015), and in the gravitropic response of lateral roots (Waidmann *et al.*, 2019).

The root system of cereals differs markedly from that of arabidopsis. Both the crown roots and lateral roots of cereals play key but different roles in maintaining plant yield. Root depth is a critical feature for productivity under non‐irrigated conditions (in which most of the world’s wheat is grown), a feature associated with crown root growth—both length and angle of the roots (Julkowska, 2018). Lateral roots form the major bulk of the root system, for both water and nutrient acquisition, and spread laterally covering a wide surface area. Recent publications are showing the critical role that the cytokinins, CKX4 in particular, play in cereal root growth, drought tolerance and the acquisition of micronutrients. Critical in this research has been the recognition of the inhibitory role that the cytokinins play in roots in contrast to the promoting role they have in shoots (Table 3).

**Table 3:** Impact of manipulating *CKX* expression in the roots on plant characteristics

Plant	Gene targeted	Alteration	Morphological response	References
Rice	*OsCKX4*	Enhancer line (↑ *OsCKX4*)	↑ crown root growth (number and length) ↓ shoot growth ↑ gravitropic response ↑ Zn in seeds ↑ yield in field ↓ cytokinin	Gao *et al. *(2014, 2019)
		Root‐specific promoter	↑ crown root growth ↑ Zn in seeds ↑ yield in field	
		RNAi	↓ crown root growth ↓ Zn	Gao *et al. * (2014)
	*OsNAC2* [transcription factor that expresses in roots]	Overexpression	↓ crown root number ↓ crown root length ↓ *CKX4* and *CKX5* ↑ cytokinin	Mao *et al. *(2019)
		RNAi and CRISPR/Cas9	↑ crown root growth	
			↑ *CKX4*	
Barley	*ZmCKX1*	Barley phosphate transporter promoter	↑ root growth ↑ tillers limited seed set	Mrízová *et al. *(2013)
	*AtCKX1*	Maize root‐specific promoter	↑ root growth ↑ drought tolerance	Pospíšilová *et al. *(2016)
	*AtCKX1* *AtCKX2*	Root‐specific promoter	↑ root length, total root surface area, root DW ↑ Zn, Fe in seeds in field trial ↑ drought tolerance No yield penalty	Ramireddy *et al. *(2018a, 2018b)
	*HvCKX1*	RNAi and CRISPR/Cas9	↑ root growth	Holubová *et al. *(2018)
	*HvCKX1*	CRISPR/Cas9	↑ root growth	Gasparis *et al. *(2019)
	*HvCKX3*	CRISPR/Cas9	↓ root growth	

Gao *et al. *(2014) showed that *OsCKX4* has a key role in the initiation of crown roots in rice. Using an enhancer line, a mutant was isolated that exhibited greater root growth and more crown roots, a stronger root gravitropic response, but reduced plant height. Molecular analysis showed that the enhancer line had enhanced expression of *OsCKX4*. Overexpression and RNAi of *OsCKX4* led to greater and lesser crown root growth, respectively. The Gao *et al. *(2014) model shows *OsCKX4* mediating the interaction between cytokinin and auxin biosynthesis. As the enhanced *OsCKX4* expression also reduced shoot growth, Gao *et al. *(2014) then linked *OsCKX4* to a root‐specific promoter and achieved enhanced crown root growth (more and longer roots) without reducing shoot growth.

Most recently, Mao *et al. *(2019) investigated the role of NAC transcription factors on root development in rice. Using both down‐regulated (RNAi and CRISPR/Cas9 lines) and overexpression of *OsNAC2*, they showed that OsNAC2 is a negative regulator of root growth, reducing both crown root number and root length. Expression of *OsIPT3* and *OsIPT5,* and *OsLOGL3* was increased, expression of *OsCKX4* and *OsCKX5* was decreased, and cytokinin levels were increased in the overexpression lines, leading to the conclusion that OsNAC2 stimulates cytokinin accumulation by repressing *CKX* expression and stimulating cytokinin biosynthesis. More specifically, this occurred through the binding of OsNAC2 to the promoter of *OsCKX4* (in addition to binding to promoters of several auxin‐related genes). The authors concluded that OsNAC2 functions as an upstream integrator of the auxin and cytokinin signals regulating the root meristem and root growth, and that its specific cytokinin target is *OsCKX4.*

Undoubtedly, an investigation of *TaCKX* gene family members is warranted in wheat as enhanced crown root growth—especially longer and lesser‐angled roots—would be of benefit in wheat grown in water‐limited environments. *TaCKX4* clusters with *OsCKX4* and is in a different sub‐clade to the *TaCKX2* cluster associated with grain yield (Fig. S1). According to the RNA‐seq database, *TaCKX4* expresses weakly in roots (Fig. S2H) including in axillary roots and in the root apical meristem (RAM) at the three‐leaf stage. However, the two *TaCKX* gene family members that express specifically in roots (*TaCKX7* and *TaCKX10*) are expressed at very low levels relative to other gene family members (Fig. S2J,M; Ogonowska *et al.*, 2019).

No comparative analysis of *TaCKXs* in crown root primordia versus lateral root primordia has been undertaken, nor has any analysis in cereals relating to the control of lateral root spacing. However, artificial constructs have been used to modify *CKX* expression and to investigate the impact on the plant of modified root growth. Transgenic barley overexpressing an arabidopsis *CKX1* gene under the control of a maize root‐specific promoter showed enhanced tolerance to drought, an effect ascribed to altered root morphology (Pospíšilová *et al.*, 2016). More recently, barley plants, transgenic for arabidopsis *CKX1* or *CKX2* under one of three root‐specific promoters (*pEPP, pPER* and *pRET*), were constructed and exhibited enhanced root systems (Ramireddy *et al.*, 2018a). Ramireddy *et al. *(2018a) selected *AtCKX1* and *AtCKX2* because they represented two lines of CKXs that diverged before the divergence of the monocots from the dicots, and because they have different subcellular localization, biochemical characteristics and substrate preferences (Schmülling *et al.*, 2003).

*CKX* expression was elevated in the roots but not the shoots, with Z‐type cytokinin conjugation reduced in the roots. Total root length, total root surface area and root dry weight were all increased in the transgenics, with no reduction in shoot biomass, while seed yield was maintained. The concentrations of several macro‐ and micronutrients were increased, especially for the *CKX2* transgenics, and this enhancement was maintained under drought conditions. In the *CKX2* transgenic lines then analysed, Ca, Cu and Zn were shown to be enhanced in the seeds.

Furthermore, the *pEPP::CKX2* transgenic lines, which showed the strongest root growth also withstood prolonged drought better (Ramireddy *et al.*, 2018a). The increased concentration of Zn is particularly significant as it is an essential element in the human diet and can be in limiting quantities when cereals are a staple food (Ramireddy *et al.*, 2018a, b). Field trials of *pEPP::CKX1* and *CKX2* plants showed not only enhanced Zn but also Fe in the seeds, along with similar grain yield and TGW indicating, significantly, that the enhanced root growth did not confer a yield penalty (Ramireddy *et al.*, 2018b). Enhanced Fe uptake is a significant feature, as previously it has been shown that cytokinin suppressed genes for Fe uptake (Séguéla *et al.*, 2008).

Andersen *et al. *(2018) show how cytokinin might influence mineral nutrient uptake in arabidopsis through investigating the formation of passage cells. These cells disrupt the suberized endodermis and facilitate water and mineral uptake. Mineral nutrient transporters have been demonstrated to be associated with passage cells (Andersen *et al.*, 2018). They suggest that endodermal cells acquire passage cell features when cytokinin is repressed. Mineral nutrient stress leads to reduced cytokinin, which may facilitate passage cell production. Likewise, enhanced CKX activity in roots should lead to enhanced passage cell production and facilitate the uptake of mineral nutrients, as was shown in the barley transgenes overexpressing CKX (Ramireddy *et al.*, 2018a, b).

Most recently, using the overexpressing *CKX4* ‘root‐enhancer’ mutant of rice, Gao *et al. *(2019) showed that reduced cytokinin elevated Zn uptake in roots and shoots under both normal and Zn‐limiting conditions. Application of cytokinin reduced Zn uptake via a reduction in the expression of the metal transporters *OsZIP1* and *OsZIP5* in root cells. They further showed that cytokinin levels were reduced under Zn deficiency. Not only was cytokinin interacting through type B RR22 to directly reduce the expression of *OsZIP1* and *OsZIP5* in the roots, but the cytokinins were shown also to be negative regulators of chelators necessary for the internal transport of Zn (Gao *et al.*, 2019). Seeds of *OsCKX4*‐overexpressing plants and mutants of the side chain‐hydroxylating enzyme that catalyses the formation of the active tZ‐type cytokinins both showed elevated Zn while those of the *Osckx2* mutant had reduced Zn concentration. Moreover, seeds from transgenic plants exhibiting root‐specific expression of *OsCKX4* had increased seed Zn (but not Fe) and, furthermore, increased overall yield when grown in the field.

This multiple action of cytokinin on Zn uptake and transport is similar to the multiple effects the cytokinins have on N uptake and assimilation, where under C limitation/excess N, cytokinin reduces N uptake while enhancing assimilation (Guo *et al.*, 2017).

The ramification of lateral roots through soil is a key aspect of the uptake of less mobile mineral nutrients. The orientation of lateral roots has generally been associated with auxin (e.g. Friml *et al.*, 2003). Lateral roots establish a distinct gravitropic set‐point angle (GSA) (Digby and Firn, 1995) at an angle to gravity that differs from the primary roots and that suggests a suppressive response to gravity (Waidmann *et al.*, 2019). Based on a genome‐wide association study (GWAS) of arabidopsis lines, Waidmann *et al. *(2019) identified a prominent peak associated with a single nucleotide polymorphism located in the *AtCKX2* gene that was associated with increased GSA values, reflecting more perpendicular LR growth to gravity. As root angle could be increased or decreased by enhancing or reducing *AtCKX2* activity, respectively, Waidmann *et al. *(2019) concluded that cytokinin signalling defines directional lateral root growth by reducing lateral root bending after emergence, through the activity of *AtCKX2*.

Various transgenic manipulations show that the cytokinins are involved in multiple different aspects of root growth and their physiology in rice and barley, but there is no information about the impact of *CKX* mutants on root growth in wheat. There is limited information about the expression of *TaCKX* gene family members in roots (Fig 4), and no information about spatial expression. As Julkowska (2018) suggests, bulk root mass may be disadvantageous when resources are limited, whereas root distribution is critical: wheat plants with lesser root angle would be ideal. As most of the world’s wheat is grown in non‐irrigated areas (Ud Dowla *et al.*, 2018), information on the control of root growth is critical.

## Future perspective

**Figure 5:**
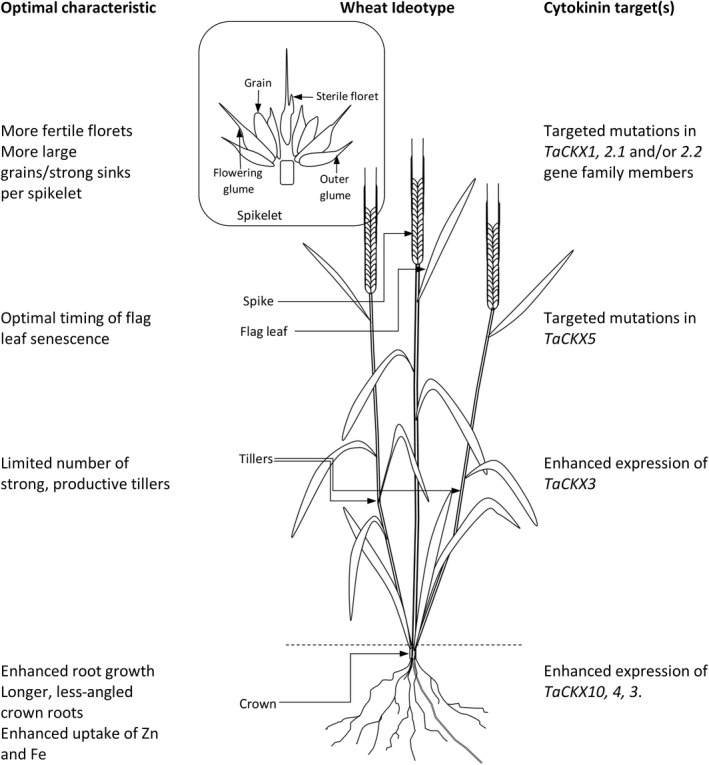
Wheat ideotype based on selected *TaCKX* mutants exhibiting increased/decreased expression in specific parts of the wheat plant.

The wheat ideotype has a limited number of strong tillers—all of which are productive—more fertile florets per spikelet and more large grains per floret, a root profile that has longer, lesser‐angled crown roots, and a flag leaf that is resilient to abiotic and biotic stressors and yet senesces at an optimum time for seed fill (Fig. 5). There is a clear divergence in *TaCKX* gene family members between those that contribute to grain yield and those that are expressed in vegetative tissues. Moreover, the contrasting effects of cytokinins on roots and shoots must be accommodated. Root growth is currently manipulated by *CKX* overexpression driven via root‐specific promoters. Reducing CKX to promote seed number by increasing spikelet number while not providing a cytokinin environment that might promote increased tillering or indeed reduced root growth is likely to be challenging. Moreover, any manipulation of the cytokinins via gene technology interventions will be further challenged by the strong homeostatic mechanisms controlling cytokinin levels in plants (Mrízová *et al.*, 2013; Ogonowska *et al.*, 2019).

While orthologues of *CKX1* and *CKX2* express in the reproductive structures of rice, barley and wheat and can be related to yield in all three cereals, other *CKX* gene family members appear to have diverged. For example, *OsCKX11* is the most strongly expressing *CKX* gene family member in the rice panicle (Yamburenko *et al.*, 2017) and yet in wheat no expression was detected in reproductive structures, and its greatest expression was in the flag leaf including the senescing blade (Fig. 4). Consequently, as previously suggested (Ninan *et al.*, 2017), it is critical to identify targets for breeding to the gene family member level in the specific species and, potentially, cultivar of interest and which, in wheat, may necessitate the identification of mutations on all three genomes. TILLING followed by whole‐exome sequencing is a mechanism by which mutations on the A, B and D genomes can be identified and subsequently stacked (Krasileva *et al.*, 2017). We suggest the most useful mutations in terms of yield enhancement are likely to be those in *TaCKX1* and the *CKX2.1* and *2.2* gene family members. In contrast, mutations in *TaCKX4, TaCKX7* and *TaCKX10* could enhance cytokinin levels in the roots but which would, in fact, be undesirable. In such cases, when gain of function of the target genes is required, mutations of the upstream regulators of the target gene could be used. For instance, Mao *et al. *(2019) were able to enhance the *OsCKX4* function in rice by down‐regulating its inactivator, NAC29.

As stated by Borrill *et al. *(2015), genomics is the key to unlocking the polyploid potential of wheat. Unlike arabidopsis, there have been very few mutations available in wheat. We have been using speed breeding (Ghosh *et al.*, 2018; Watson *et al.*, 2018) and whole‐exome capture of a TILLING population of Jimai22 to overcome this limitation. Jimai22 is the most popular cultivar of wheat currently grown across the major wheat‐producing regions in China. Our aim is to sequence over 1000 mutant lines to develop a hexaploid wheat mutant gene bank containing over one million missense and stop codon mutation sites covering 99% of the high‐confidence genes in the IWGSC RefSeq v2.0. We plan to identify and functionally characterize beneficial gene alleles, focusing initially on cytokinin regulatory genes including *TaCKX* gene family members. Our initial database, containing over 770,000 EST‐type mutants in 94,900 high‐confidence genes, is now available online (://www.ytjebc.com). So far, we have identified multiple missense/stop codon mutant alleles in each of the A, B and D sub‐genomes for all the *TaCKX* gene family members. Functional analysis of these mutants is currently underway.

## Conflicts of interest

The authors declare they have no conflicts of interest.

## Author contributions

L.C. interrogated the wheat genome databases and prepared the gene position and expression figures; J.S. and J.Z. interrogated the phylogenetic data and prepared the relevant figures; and P.E.J. wrote the first draft of the review with input subsequently from all authors.

## Supporting information


**Figure S1** Phylogenetic cladogram showing the basis of the *TaCKX* naming.

**Figure S2** RNA‐seq graphs for all *TaCKX* gene family members.
