# Supplementary material for: Cytokinin dehydrogenase: a genetic target for yield improvement in wheat
Source: Plant Biotechnol J. 2019 Dec 22;18(3):614–30. doi: 10.1111/pbi.13305 (PMC7004901; doi:10.1111/pbi.13305)
Supplement: Supplementary file 1 — Figure S1 Phylogenetic cladogram showing the basis of the TaCKX naming. [file PBI-18-614-s001.pdf]

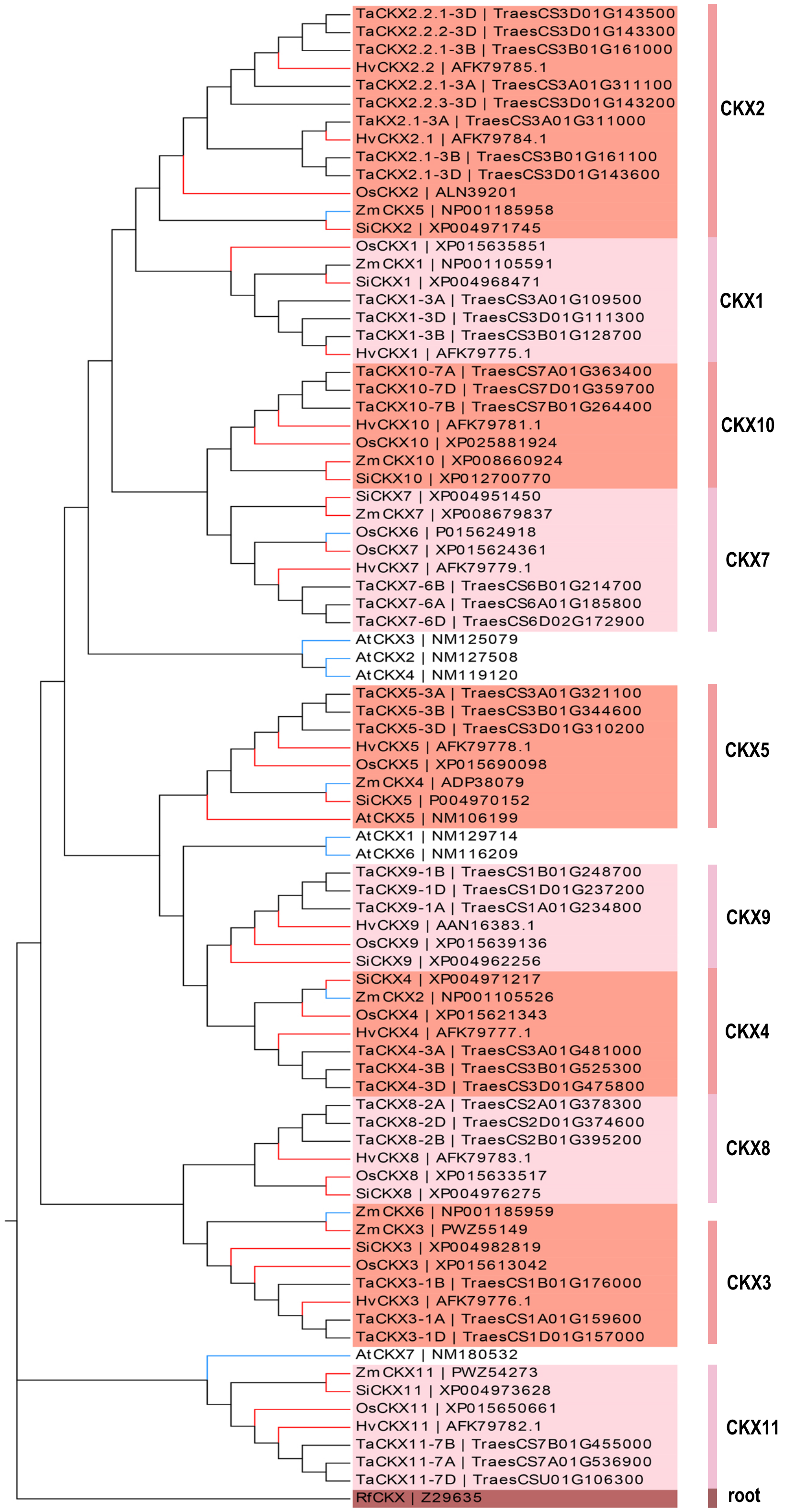

Fig S1. Phylogenetic cladogram showing the basis of the *TaCKX* naming. Tomato and pink colours show the separation of different *TaCKX* subfamilies. Red branch lines indicate consistent gene numbers from other species corresponding to *Triticum aestivum*, while blue branch lines indicate non-consistent gene numbers. Neighbor Joining phylogenetic tree for CKX proteins in *Triticum aestivum* (Ta), *Hordeum vulgare* (Hv)*, Oryza sativa* (Os)*, Zea mays* (Zm), *Setaria italica* (Si) and *Arabidopsis thaliana* (At). The tree was rooted using CKX protein from *Rhodococcus fascians* (Rf). Node values are percentages of bootstraps generated with 1000 bootstrap replicates. Protein data were collected from NCBI database. Neighbor Joining phylogenetic tree generated by MEGA-X. The phylogenetic cladogram was edited in EvolView 2 (He et al. 2016; Zhang et al. 2012).
