# Supplementary material for: Cytokinin dehydrogenase: a genetic target for yield improvement in wheat
Source: Plant Biotechnol J. 2019 Dec 22;18(3):614–30. doi: 10.1111/pbi.13305 (PMC7004901; doi:10.1111/pbi.13305)
Supplement: Supplementary file 2 — Figure S2 RNA‐seq graphs for all TaCKX gene family members. [file PBI-18-614-s002.pdf]

A.

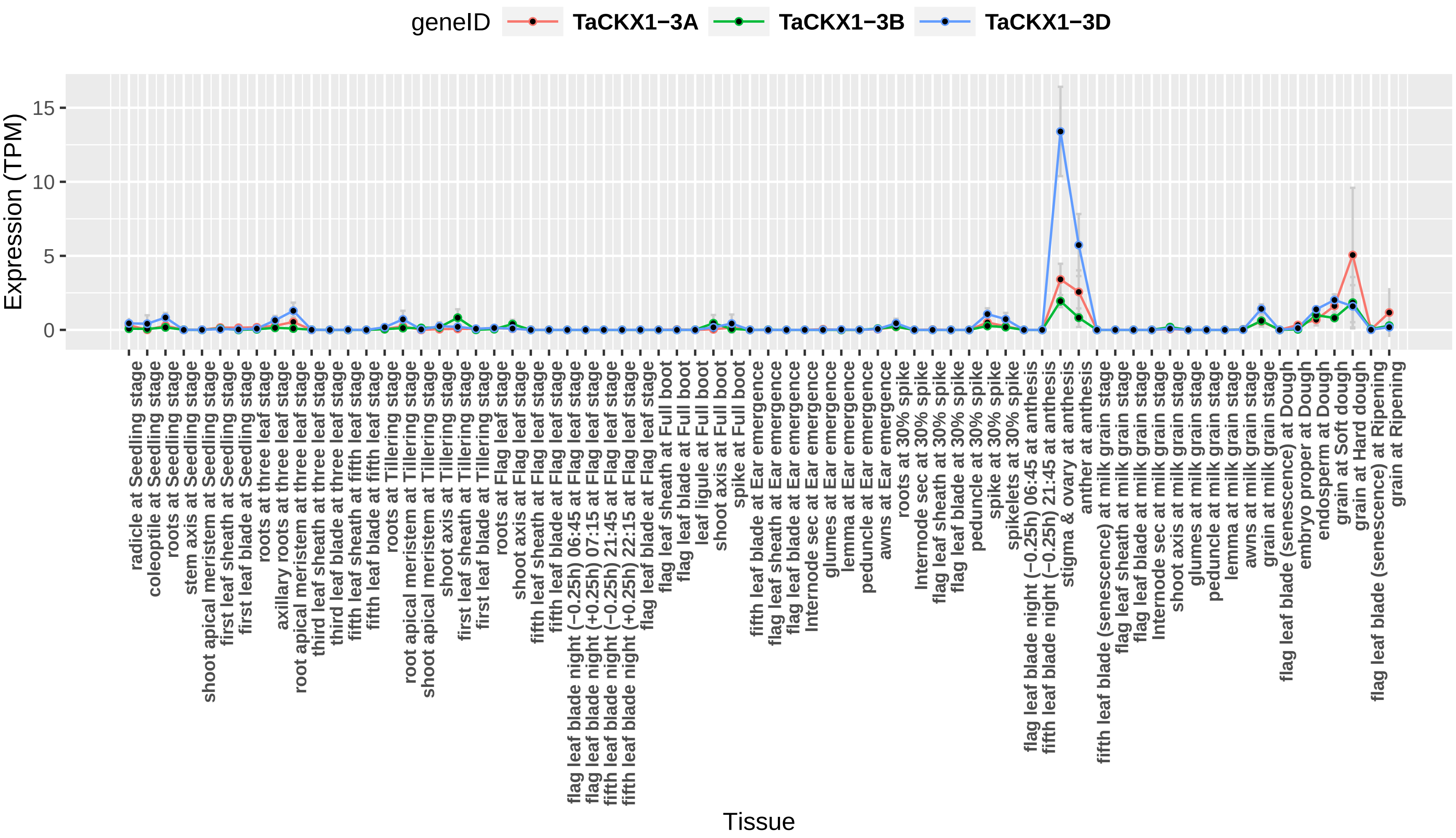

geneID
TaCKX1−3A
TaCKX1−3B
TaCKX1−3D
Expression (TPM)
0
5
10
15
radicle at Seedling stage
coleoptile at Seedling stage
roots at Seedling stage
stem axis at Seedling stage
shoot apical meristem at Seedling stage
first leaf sheath at Seedling stage
first leaf blade at Seedling stage
roots at three leaf stage
axillary roots at three leaf stage
root apical meristem at three leaf stage
third leaf sheath at three leaf stage
third leaf blade at three leaf stage
fifth leaf sheath at fifth leaf stage
fifth leaf blade at fifth leaf stage
roots at Tillering stage
root apical meristem at Tillering stage
shoot apical meristem at Tillering stage
shoot axis at Tillering stage
first leaf sheath at Tillering stage
first leaf blade at Tillering stage
roots at Flag leaf stage
shoot axis at Flag leaf stage
fifth leaf sheath at Flag leaf stage
fifth leaf blade at Flag leaf stage
flag leaf blade night (−0.25h) 06:45 at Flag leaf stage
flag leaf blade night (+0.25h) 07:15 at Flag leaf stage
fifth leaf blade night (−0.25h) 21:45 at Flag leaf stage
fifth leaf blade night (+0.25h) 22:15 at Flag leaf stage
flag leaf blade at Flag leaf stage
flag leaf sheath at Full boot
flag leaf blade at Full boot
leaf ligule at Full boot
shoot axis at Full boot
spike at Full boot
fifth leaf blade at Ear emergence
flag leaf sheath at Ear emergence
flag leaf blade at Ear emergence
Internode sec at Ear emergence
glumes at Ear emergence
lemma at Ear emergence
peduncle at Ear emergence
awns at Ear emergence
roots at 30% spike
Internode sec at 30% spike
flag leaf sheath at 30% spike
flag leaf blade at 30% spike
peduncle at 30% spike
spike at 30% spike
spikelets at 30% spike
flag leaf blade night (−0.25h) 06:45 at anthesis
fifth leaf blade night (−0.25h) 21:45 at anthesis
stigma & ovary at anthesis
anther at anthesis
fifth leaf blade (senescence) at milk grain stage
flag leaf sheath at milk grain stage
flag leaf blade at milk grain stage
Internode sec at milk grain stage
shoot axis at milk grain stage
glumes at milk grain stage
peduncle at milk grain stage
lemma at milk grain stage
awns at milk grain stage
grain at milk grain stage
flag leaf blade (senescence) at Dough
embryo proper at Dough
endosperm at Dough
grain at Soft dough
grain at Hard dough
flag leaf blade (senescence) at Ripening
grain at Ripening
Tissue

B.

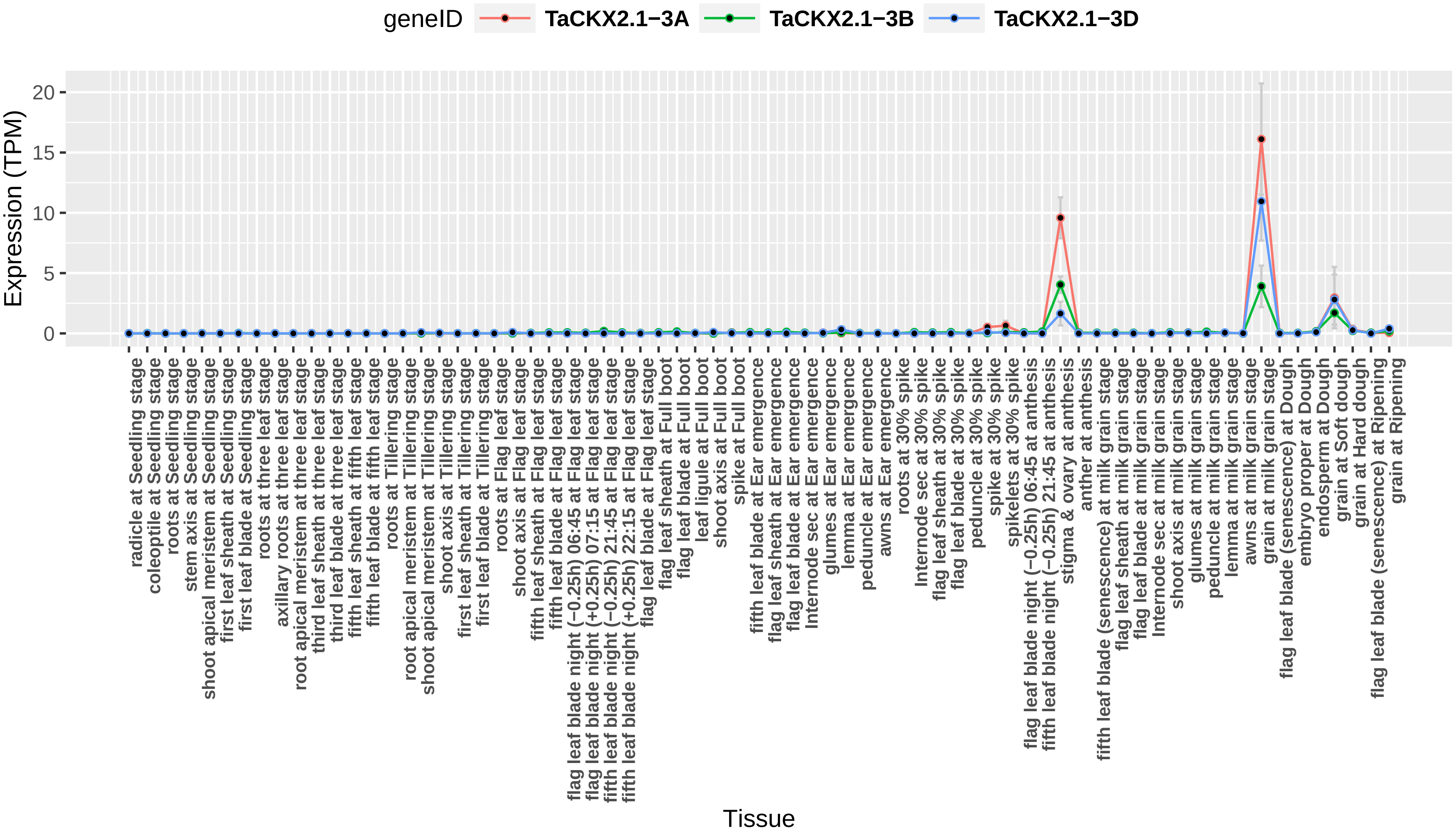
geneID
TaCKX2.1−3A
TaCKX2.1−3B
TaCKX2.1−3D
Expression (TPM)
0
5
10
15
20
Tissue
radicle at Seedling stage
coleoptile at Seedling stage
roots at Seedling stage
stem axis at Seedling stage
shoot apical meristem at Seedling stage
first leaf sheath at Seedling stage
first leaf blade at Seedling stage
roots at three leaf stage
axillary roots at three leaf stage
root apical meristem at three leaf stage
third leaf sheath at three leaf stage
third leaf blade at three leaf stage
fifth leaf sheath at fifth leaf stage
fifth leaf blade at fifth leaf stage
roots at Tillering stage
root apical meristem at Tillering stage
shoot apical meristem at Tillering stage
shoot axis at Tillering stage
first leaf sheath at Tillering stage
first leaf blade at Tillering stage
roots at Flag leaf stage
shoot axis at Flag leaf stage
fifth leaf sheath at Flag leaf stage
fifth leaf blade at Flag leaf stage
flag leaf blade night (−0.25h) 06:45 at Flag leaf stage
flag leaf blade night (+0.25h) 07:15 at Flag leaf stage
fifth leaf blade night (−0.25h) 21:45 at Flag leaf stage
fifth leaf blade night (+0.25h) 22:15 at Flag leaf stage
flag leaf blade at Flag leaf stage
flag leaf sheath at Full boot
flag leaf blade at Full boot
leaf ligule at Full boot
shoot axis at Full boot
spike at Full boot
fifth leaf blade at Ear emergence
flag leaf sheath at Ear emergence
flag leaf blade at Ear emergence
Internode sec at Ear emergence
glumes at Ear emergence
lemma at Ear emergence
peduncle at Ear emergence
awns at Ear emergence
roots at 30% spike
Internode sec at 30% spike
flag leaf sheath at 30% spike
flag leaf blade at 30% spike
peduncle at 30% spike
spike at 30% spike
spikelets at 30% spike
flag leaf blade night (−0.25h) 06:45 at anthesis
fifth leaf blade night (−0.25h) 21:45 at anthesis
stigma & ovary at anthesis
anther at anthesis
fifth leaf blade (senescence) at milk grain stage
flag leaf sheath at milk grain stage
flag leaf blade at milk grain stage
Internode sec at milk grain stage
shoot axis at milk grain stage
glumes at milk grain stage
peduncle at milk grain stage
lemma at milk grain stage
awns at milk grain stage
grain at milk grain stage
flag leaf blade (senescence) at Dough
embryo proper at Dough
endosperm at Dough
grain at Soft dough
grain at Hard dough
flag leaf blade (senescence) at Ripening
grain at Ripening

C.

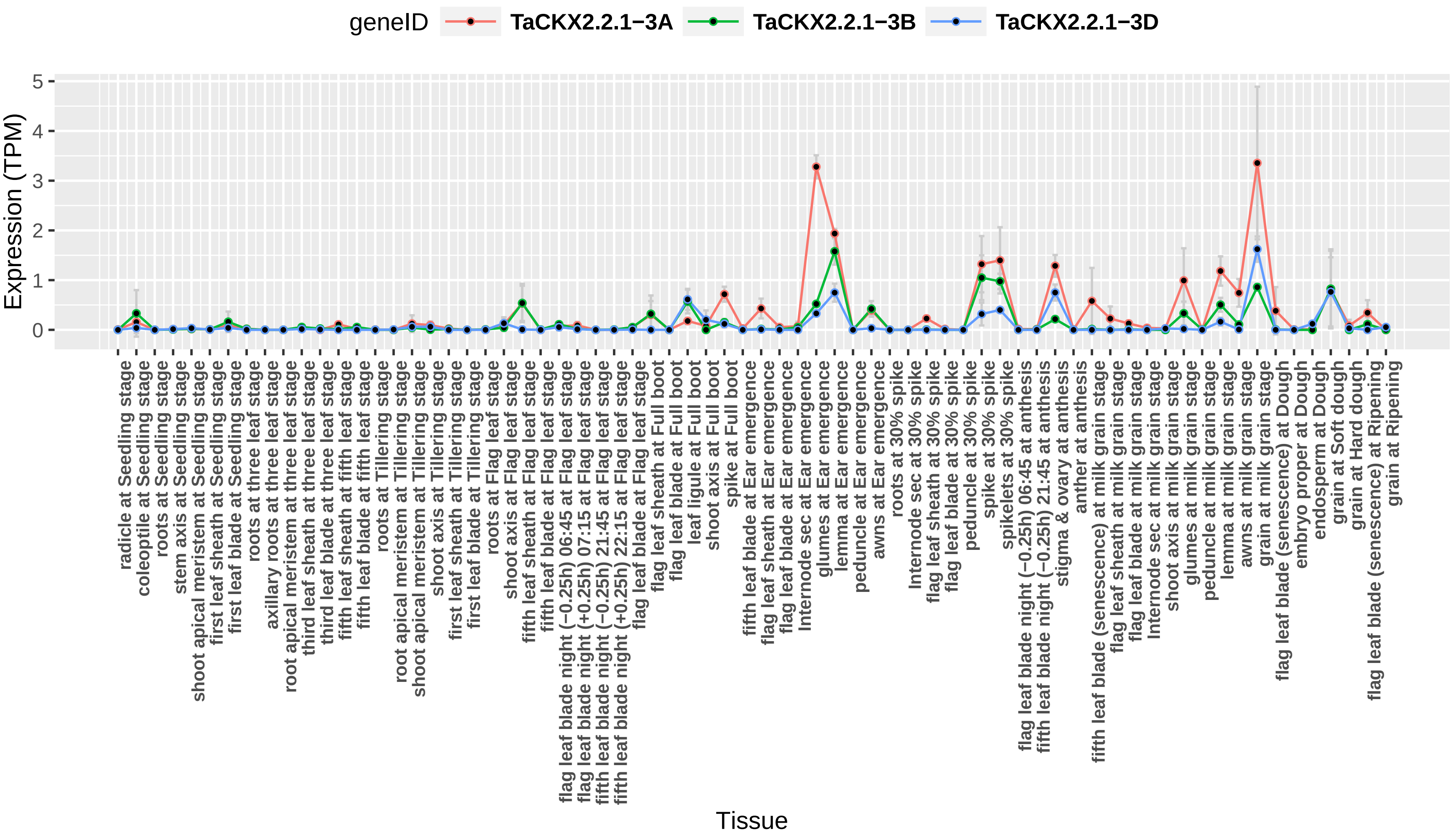

geneID
TaCKX2.2.1−3A
TaCKX2.2.1−3B
TaCKX2.2.1−3D
Expression (TPM)
0
1
2
3
4
5
Tissue
radicle at Seedling stage
coleoptile at Seedling stage
roots at Seedling stage
stem axis at Seedling stage
shoot apical meristem at Seedling stage
first leaf sheath at Seedling stage
first leaf blade at Seedling stage
roots at three leaf stage
axillary roots at three leaf stage
root apical meristem at three leaf stage
third leaf sheath at three leaf stage
third leaf blade at three leaf stage
fifth leaf sheath at fifth leaf stage
fifth leaf blade at fifth leaf stage
roots at Tillering stage
root apical meristem at Tillering stage
shoot apical meristem at Tillering stage
shoot axis at Tillering stage
first leaf sheath at Tillering stage
first leaf blade at Tillering stage
roots at Flag leaf stage
shoot axis at Flag leaf stage
fifth leaf sheath at Flag leaf stage
fifth leaf blade at Flag leaf stage
flag leaf blade night (−0.25h) 06:45 at Flag leaf stage
flag leaf blade night (+0.25h) 07:15 at Flag leaf stage
fifth leaf blade night (−0.25h) 21:45 at Flag leaf stage
fifth leaf blade night (+0.25h) 22:15 at Flag leaf stage
flag leaf blade at Flag leaf stage
flag leaf sheath at Full boot
flag leaf blade at Full boot
leaf ligule at Full boot
shoot axis at Full boot
spike at Full boot
fifth leaf blade at Ear emergence
flag leaf sheath at Ear emergence
flag leaf blade at Ear emergence
Internode sec at Ear emergence
glumes at Ear emergence
lemma at Ear emergence
peduncle at Ear emergence
awns at Ear emergence
roots at 30% spike
Internode sec at 30% spike
flag leaf sheath at 30% spike
flag leaf blade at 30% spike
peduncle at 30% spike
spike at 30% spike
spikelets at 30% spike
flag leaf blade night (−0.25h) 06:45 at anthesis
fifth leaf blade night (−0.25h) 21:45 at anthesis
stigma & ovary at anthesis
anther at anthesis
fifth leaf blade (senescence) at milk grain stage
flag leaf sheath at milk grain stage
flag leaf blade at milk grain stage
Internode sec at milk grain stage
shoot axis at milk grain stage
glumes at milk grain stage
peduncle at milk grain stage
lemma at milk grain stage
awns at milk grain stage
grain at milk grain stage
flag leaf blade (senescence) at Dough
embryo proper at Dough
endosperm at Dough
grain at Soft dough
grain at Hard dough
flag leaf blade (senescence) at Ripening
grain at Ripening

D.

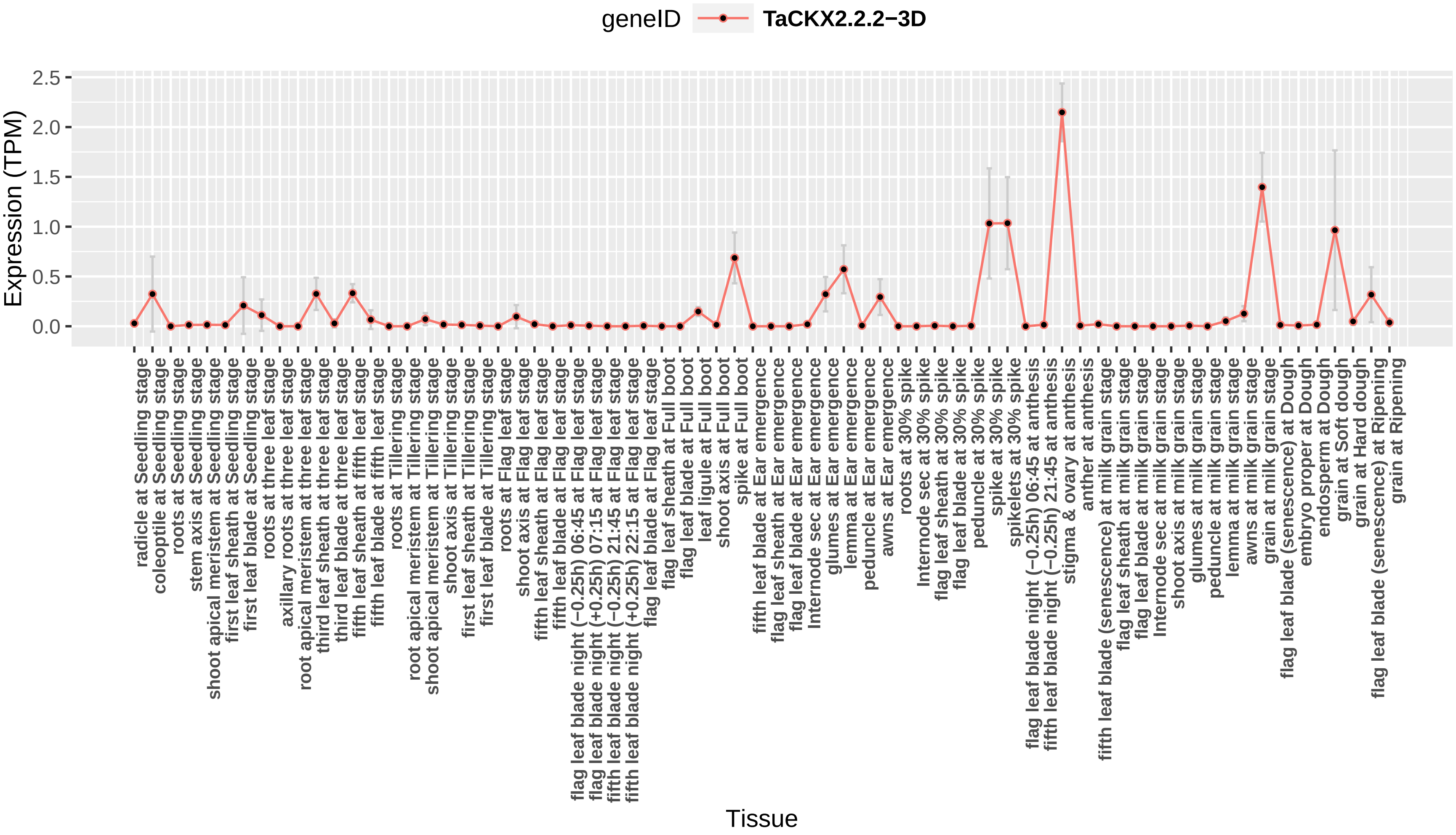
geneID
TaCKX2.2.2−3D
Expression (TPM)
2.5
2.0
1.5
1.0
0.5
0.0
radicle at Seedling stage
coleoptile at Seedling stage
roots at Seedling stage
stem axis at Seedling stage
shoot apical meristem at Seedling stage
first leaf sheath at Seedling stage
first leaf blade at Seedling stage
roots at three leaf stage
axillary roots at three leaf stage
root apical meristem at three leaf stage
third leaf sheath at three leaf stage
third leaf blade at three leaf stage
fifth leaf sheath at fifth leaf stage
fifth leaf blade at fifth leaf stage
roots at Tillering stage
root apical meristem at Tillering stage
shoot apical meristem at Tillering stage
shoot axis at Tillering stage
first leaf sheath at Tillering stage
first leaf blade at Tillering stage
roots at Flag leaf stage
shoot axis at Flag leaf stage
fifth leaf sheath at Flag leaf stage
fifth leaf blade at Flag leaf stage
flag leaf blade night (−0.25h) 06:45 at Flag leaf stage
flag leaf blade night (+0.25h) 07:15 at Flag leaf stage
fifth leaf blade night (−0.25h) 21:45 at Flag leaf stage
fifth leaf blade night (+0.25h) 22:15 at Flag leaf stage
flag leaf blade at Flag leaf stage
flag leaf sheath at Full boot
flag leaf blade at Full boot
leaf ligule at Full boot
shoot axis at Full boot
spike at Full boot
fifth leaf blade at Ear emergence
flag leaf sheath at Ear emergence
flag leaf blade at Ear emergence
Internode sec at Ear emergence
glumes at Ear emergence
lemma at Ear emergence
peduncle at Ear emergence
awns at Ear emergence
roots at 30% spike
Internode sec at 30% spike
flag leaf sheath at 30% spike
flag leaf blade at 30% spike
peduncle at 30% spike
spike at 30% spike
spikelets at 30% spike
flag leaf blade night (−0.25h) 06:45 at anthesis
fifth leaf blade night (−0.25h) 21:45 at anthesis
stigma & ovary at anthesis
anther at anthesis
fifth leaf blade (senescence) at milk grain stage
flag leaf sheath at milk grain stage
flag leaf blade at milk grain stage
Internode sec at milk grain stage
shoot axis at milk grain stage
glumes at milk grain stage
peduncle at milk grain stage
lemma at milk grain stage
awns at milk grain stage
grain at milk grain stage
flag leaf blade (senescence) at Dough
embryo proper at Dough
endosperm at Dough
grain at Soft dough
grain at Hard dough
flag leaf blade (senescence) at Ripening
grain at Ripening
Tissue

E.

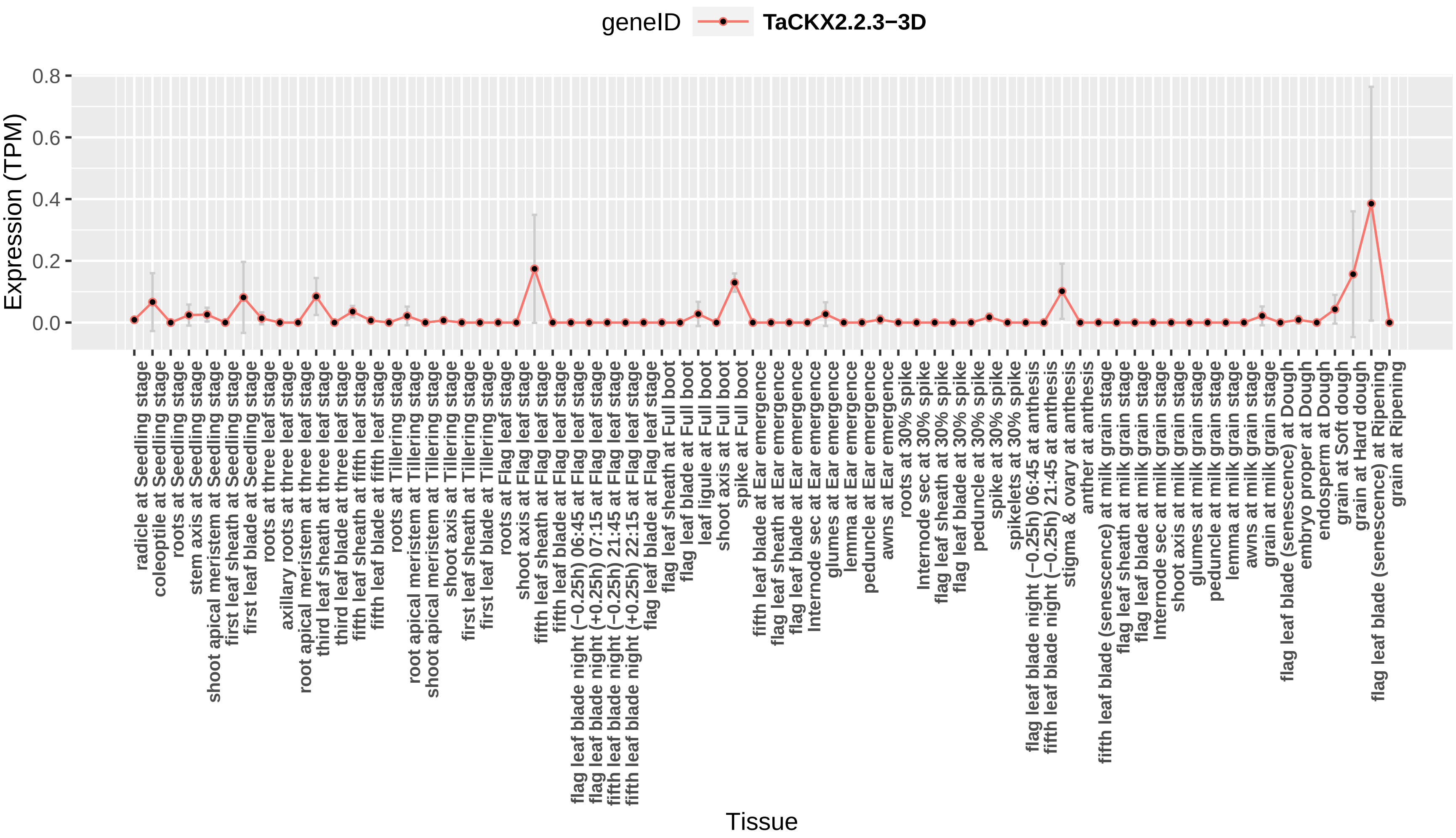
geneID
TaCKX2.2.3−3D
Expression (TPM)
0.8
0.6
0.4
0.2
0.0
Tissue
radicle at Seedling stage
coleoptile at Seedling stage
roots at Seedling stage
stem axis at Seedling stage
shoot apical meristem at Seedling stage
first leaf sheath at Seedling stage
first leaf blade at Seedling stage
roots at three leaf stage
axillary roots at three leaf stage
root apical meristem at three leaf stage
third leaf sheath at three leaf stage
third leaf blade at three leaf stage
fifth leaf sheath at fifth leaf stage
fifth leaf blade at fifth leaf stage
roots at Tillering stage
root apical meristem at Tillering stage
shoot apical meristem at Tillering stage
shoot axis at Tillering stage
first leaf sheath at Tillering stage
first leaf blade at Tillering stage
roots at Flag leaf stage
shoot axis at Flag leaf stage
fifth leaf sheath at Flag leaf stage
fifth leaf blade at Flag leaf stage
flag leaf blade night (−0.25h) 06:45 at Flag leaf stage
flag leaf blade night (+0.25h) 07:15 at Flag leaf stage
fifth leaf blade night (−0.25h) 21:45 at Flag leaf stage
fifth leaf blade night (+0.25h) 22:15 at Flag leaf stage
flag leaf blade at Flag leaf stage
flag leaf sheath at Full boot
flag leaf blade at Full boot
leaf ligule at Full boot
shoot axis at Full boot
spike at Full boot
fifth leaf blade at Ear emergence
flag leaf sheath at Ear emergence
flag leaf blade at Ear emergence
Internode sec at Ear emergence
glumes at Ear emergence
lemma at Ear emergence
peduncle at Ear emergence
awns at Ear emergence
roots at 30% spike
Internode sec at 30% spike
flag leaf sheath at 30% spike
flag leaf blade at 30% spike
peduncle at 30% spike
spike at 30% spike
spikelets at 30% spike
flag leaf blade night (−0.25h) 06:45 at anthesis
fifth leaf blade night (−0.25h) 21:45 at anthesis
stigma & ovary at anthesis
anther at anthesis
fifth leaf blade (senescence) at milk grain stage
flag leaf sheath at milk grain stage
flag leaf blade at milk grain stage
Internode sec at milk grain stage
shoot axis at milk grain stage
glumes at milk grain stage
peduncle at milk grain stage
lemma at milk grain stage
awns at milk grain stage
grain at milk grain stage
flag leaf blade (senescence) at Dough
embryo proper at Dough
endosperm at Dough
grain at Soft dough
grain at Hard dough
flag leaf blade (senescence) at Ripening
grain at Ripening

F.

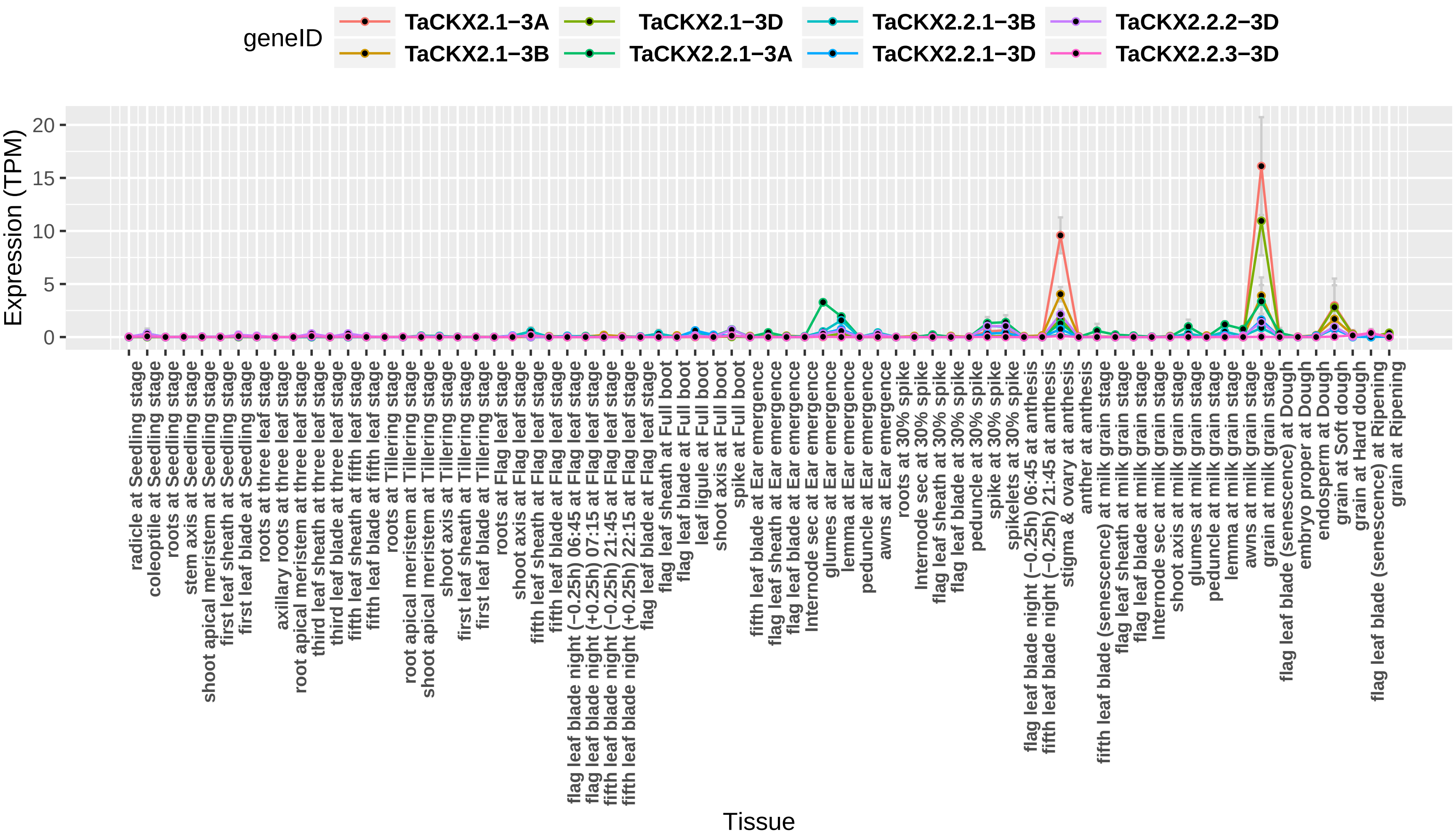

geneID
TaCKX2.1−3A
TaCKX2.1−3B
TaCKX2.1−3D
TaCKX2.2.1−3A
TaCKX2.2.1−3B
TaCKX2.2.1−3D
TaCKX2.2.2−3D
TaCKX2.2.3−3D
Expression (TPM)
20
15
10
5
0
radicle at Seedling stage
coleoptile at Seedling stage
roots at Seedling stage
stem axis at Seedling stage
shoot apical meristem at Seedling stage
first leaf sheath at Seedling stage
first leaf blade at Seedling stage
roots at three leaf stage
axillary roots at three leaf stage
root apical meristem at three leaf stage
third leaf sheath at three leaf stage
third leaf blade at three leaf stage
fifth leaf sheath at fifth leaf stage
fifth leaf blade at fifth leaf stage
roots at Tillering stage
root apical meristem at Tillering stage
shoot apical meristem at Tillering stage
shoot axis at Tillering stage
first leaf sheath at Tillering stage
first leaf blade at Tillering stage
roots at Flag leaf stage
shoot axis at Flag leaf stage
fifth leaf sheath at Flag leaf stage
fifth leaf blade at Flag leaf stage
flag leaf blade night (−0.25h) 06:45 at Flag leaf stage
flag leaf blade night (+0.25h) 07:15 at Flag leaf stage
fifth leaf blade night (−0.25h) 21:45 at Flag leaf stage
fifth leaf blade night (+0.25h) 22:15 at Flag leaf stage
flag leaf blade at Flag leaf stage
flag leaf sheath at Full boot
flag leaf blade at Full boot
leaf ligule at Full boot
shoot axis at Full boot
spike at Full boot
fifth leaf blade at Ear emergence
flag leaf sheath at Ear emergence
flag leaf blade at Ear emergence
Internode sec at Ear emergence
glumes at Ear emergence
lemma at Ear emergence
peduncle at Ear emergence
awns at Ear emergence
roots at 30% spike
Internode sec at 30% spike
flag leaf sheath at 30% spike
flag leaf blade at 30% spike
peduncle at 30% spike
spike at 30% spike
spikelets at 30% spike
flag leaf blade night (−0.25h) 06:45 at anthesis
fifth leaf blade night (−0.25h) 21:45 at anthesis
stigma & ovary at anthesis
anther at anthesis
fifth leaf blade (senescence) at milk grain stage
flag leaf sheath at milk grain stage
flag leaf blade at milk grain stage
Internode sec at milk grain stage
shoot axis at milk grain stage
glumes at milk grain stage
peduncle at milk grain stage
lemma at milk grain stage
awns at milk grain stage
grain at milk grain stage
flag leaf blade (senescence) at Dough
embryo proper at Dough
endosperm at Dough
grain at Soft dough
grain at Hard dough
flag leaf blade (senescence) at Ripening
grain at Ripening
Tissue

G.

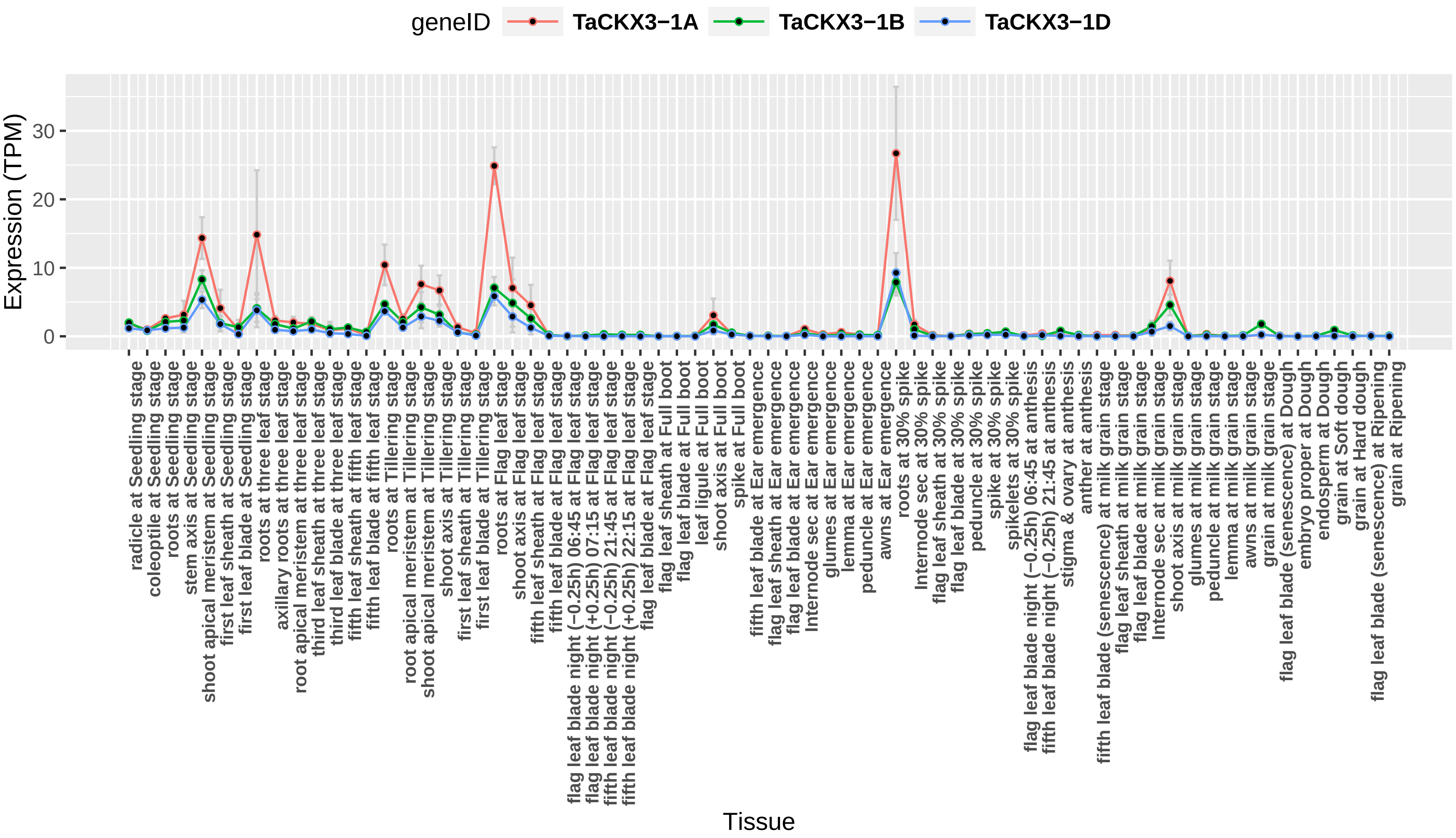
geneID
TaCKX3−1A
TaCKX3−1B
TaCKX3−1D
Expression (TPM)
0
10
20
30
Tissue
radicle at Seedling stage
coleoptile at Seedling stage
roots at Seedling stage
stem axis at Seedling stage
shoot apical meristem at Seedling stage
first leaf sheath at Seedling stage
first leaf blade at Seedling stage
roots at three leaf stage
axillary roots at three leaf stage
root apical meristem at three leaf stage
third leaf sheath at three leaf stage
third leaf blade at three leaf stage
fifth leaf sheath at fifth leaf stage
fifth leaf blade at fifth leaf stage
roots at Tillering stage
root apical meristem at Tillering stage
shoot apical meristem at Tillering stage
shoot axis at Tillering stage
first leaf sheath at Tillering stage
first leaf blade at Tillering stage
roots at Flag leaf stage
shoot axis at Flag leaf stage
fifth leaf sheath at Flag leaf stage
fifth leaf blade at Flag leaf stage
flag leaf blade night (−0.25h) 06:45 at Flag leaf stage
flag leaf blade night (+0.25h) 07:15 at Flag leaf stage
fifth leaf blade night (−0.25h) 21:45 at Flag leaf stage
fifth leaf blade night (+0.25h) 22:15 at Flag leaf stage
flag leaf blade at Flag leaf stage
flag leaf sheath at Full boot
flag leaf blade at Full boot
leaf ligule at Full boot
shoot axis at Full boot
spike at Full boot
fifth leaf blade at Ear emergence
flag leaf sheath at Ear emergence
flag leaf blade at Ear emergence
Internode sec at Ear emergence
glumes at Ear emergence
lemma at Ear emergence
peduncle at Ear emergence
awns at Ear emergence
roots at 30% spike
Internode sec at 30% spike
flag leaf sheath at 30% spike
flag leaf blade at 30% spike
peduncle at 30% spike
spike at 30% spike
spikelets at 30% spike
flag leaf blade night (−0.25h) 06:45 at anthesis
fifth leaf blade night (−0.25h) 21:45 at anthesis
stigma & ovary at anthesis
anther at anthesis
fifth leaf blade (senescence) at milk grain stage
flag leaf sheath at milk grain stage
flag leaf blade at milk grain stage
Internode sec at milk grain stage
shoot axis at milk grain stage
glumes at milk grain stage
peduncle at milk grain stage
lemma at milk grain stage
awns at milk grain stage
grain at milk grain stage
flag leaf blade (senescence) at Dough
embryo proper at Dough
endosperm at Dough
grain at Soft dough
grain at Hard dough
flag leaf blade (senescence) at Ripening
grain at Ripening

H.

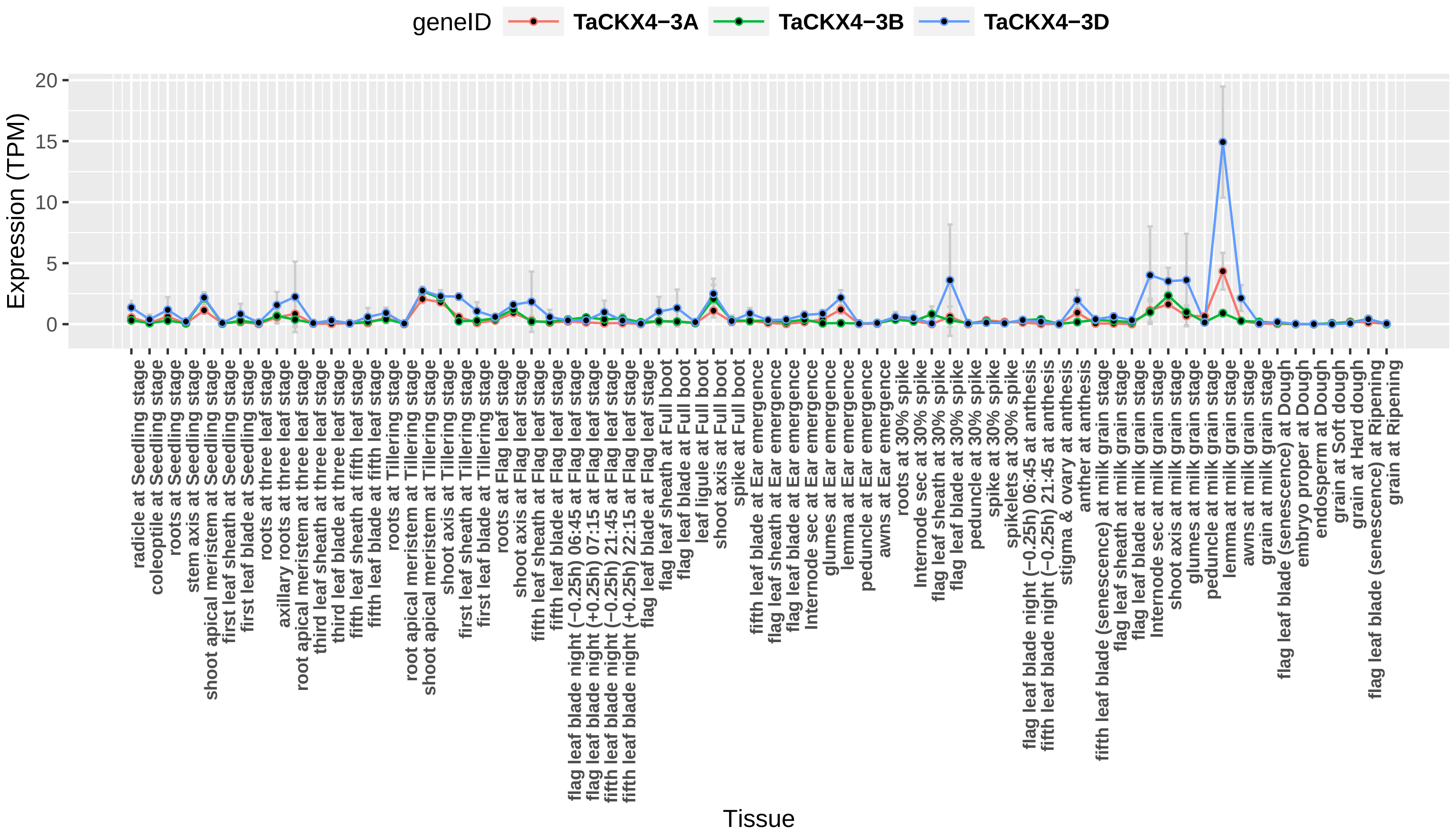

geneID
TaCKX4−3A
TaCKX4−3B
TaCKX4−3D
Expression (TPM)
20
15
10
5
0
radicle at Seedling stage
coleoptile at Seedling stage
roots at Seedling stage
stem axis at Seedling stage
shoot apical meristem at Seedling stage
first leaf sheath at Seedling stage
first leaf blade at Seedling stage
roots at three leaf stage
axillary roots at three leaf stage
root apical meristem at three leaf stage
third leaf sheath at three leaf stage
third leaf blade at three leaf stage
fifth leaf sheath at fifth leaf stage
fifth leaf blade at fifth leaf stage
roots at Tillering stage
root apical meristem at Tillering stage
shoot apical meristem at Tillering stage
shoot axis at Tillering stage
first leaf sheath at Tillering stage
first leaf blade at Tillering stage
roots at Flag leaf stage
shoot axis at Flag leaf stage
fifth leaf sheath at Flag leaf stage
fifth leaf blade at Flag leaf stage
flag leaf blade night (−0.25h) 06:45 at Flag leaf stage
flag leaf blade night (+0.25h) 07:15 at Flag leaf stage
fifth leaf blade night (−0.25h) 21:45 at Flag leaf stage
fifth leaf blade night (+0.25h) 22:15 at Flag leaf stage
flag leaf blade at Flag leaf stage
flag leaf sheath at Full boot
flag leaf blade at Full boot
leaf ligule at Full boot
shoot axis at Full boot
spike at Full boot
fifth leaf blade at Ear emergence
flag leaf sheath at Ear emergence
flag leaf blade at Ear emergence
Internode sec at Ear emergence
glumes at Ear emergence
lemma at Ear emergence
peduncle at Ear emergence
awns at Ear emergence
roots at 30% spike
Internode sec at 30% spike
flag leaf sheath at 30% spike
flag leaf blade at 30% spike
peduncle at 30% spike
spike at 30% spike
spikelets at 30% spike
flag leaf blade night (−0.25h) 06:45 at anthesis
fifth leaf blade night (−0.25h) 21:45 at anthesis
stigma & ovary at anthesis
anther at anthesis
fifth leaf blade (senescence) at milk grain stage
flag leaf sheath at milk grain stage
flag leaf blade at milk grain stage
Internode sec at milk grain stage
shoot axis at milk grain stage
glumes at milk grain stage
peduncle at milk grain stage
lemma at milk grain stage
awns at milk grain stage
grain at milk grain stage
flag leaf blade (senescence) at Dough
embryo proper at Dough
endosperm at Dough
grain at Soft dough
grain at Hard dough
flag leaf blade (senescence) at Ripening
grain at Ripening
Tissue

I.

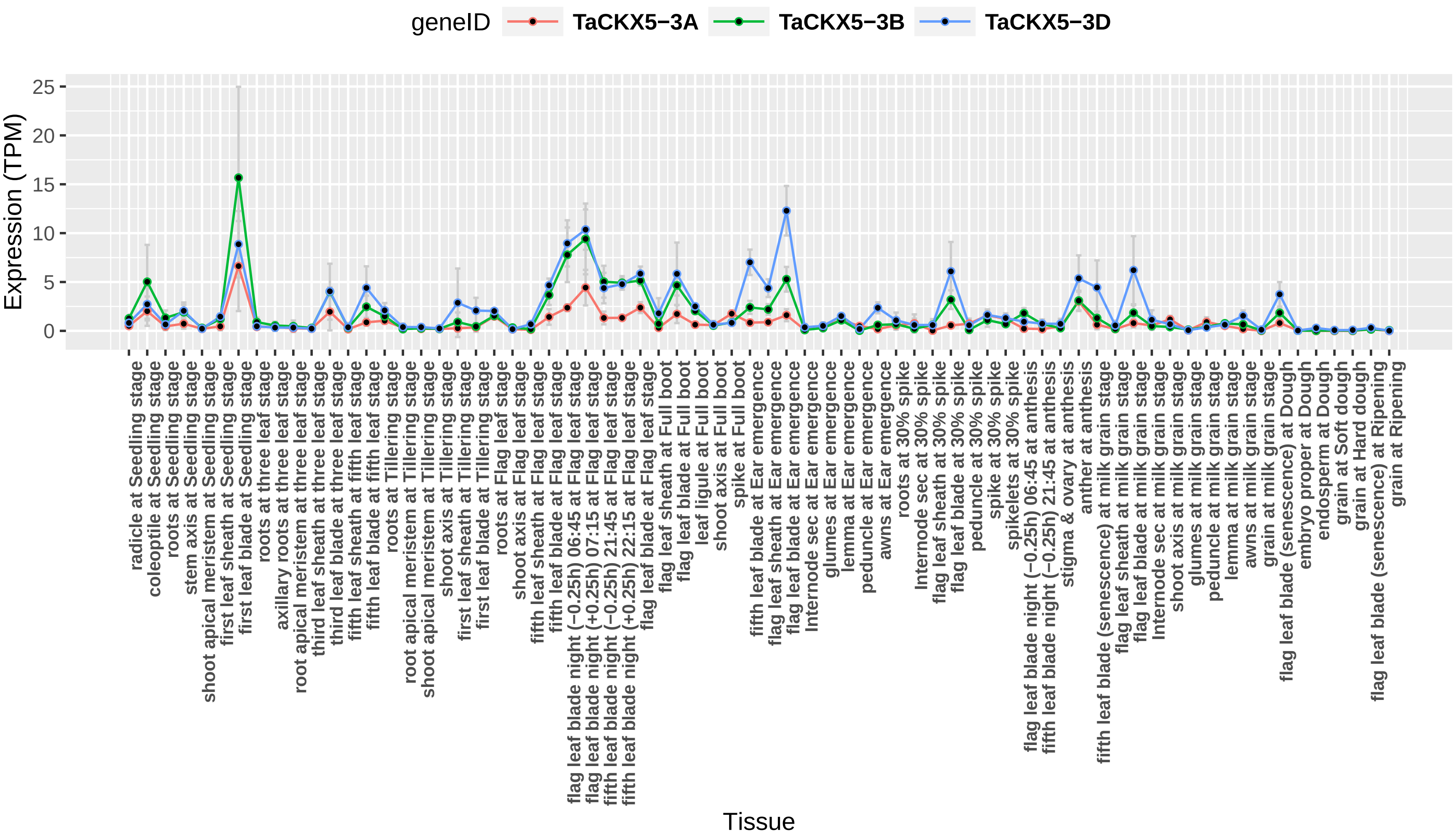

geneID
TaCKX5−3A
TaCKX5−3B
TaCKX5−3D
Expression (TPM)
25
20
15
10
5
0
radicle at Seedling stage
coleoptile at Seedling stage
roots at Seedling stage
stem axis at Seedling stage
shoot apical meristem at Seedling stage
first leaf sheath at Seedling stage
first leaf blade at Seedling stage
roots at three leaf stage
axillary roots at three leaf stage
root apical meristem at three leaf stage
third leaf sheath at three leaf stage
third leaf blade at three leaf stage
fifth leaf sheath at fifth leaf stage
fifth leaf blade at fifth leaf stage
roots at Tillering stage
root apical meristem at Tillering stage
shoot apical meristem at Tillering stage
shoot axis at Tillering stage
first leaf sheath at Tillering stage
first leaf blade at Tillering stage
roots at Flag leaf stage
shoot axis at Flag leaf stage
fifth leaf sheath at Flag leaf stage
fifth leaf blade at Flag leaf stage
flag leaf blade night (−0.25h) 06:45 at Flag leaf stage
flag leaf blade night (+0.25h) 07:15 at Flag leaf stage
fifth leaf blade night (−0.25h) 21:45 at Flag leaf stage
fifth leaf blade night (+0.25h) 22:15 at Flag leaf stage
flag leaf blade at Flag leaf stage
flag leaf sheath at Full boot
flag leaf blade at Full boot
leaf ligule at Full boot
shoot axis at Full boot
spike at Full boot
fifth leaf blade at Ear emergence
flag leaf sheath at Ear emergence
flag leaf blade at Ear emergence
Internode sec at Ear emergence
glumes at Ear emergence
lemma at Ear emergence
peduncle at Ear emergence
awns at Ear emergence
roots at 30% spike
Internode sec at 30% spike
flag leaf sheath at 30% spike
flag leaf blade at 30% spike
peduncle at 30% spike
spike at 30% spike
spikelets at 30% spike
flag leaf blade night (−0.25h) 06:45 at anthesis
fifth leaf blade night (−0.25h) 21:45 at anthesis
stigma & ovary at anthesis
anther at anthesis
fifth leaf blade (senescence) at milk grain stage
flag leaf sheath at milk grain stage
flag leaf blade at milk grain stage
Internode sec at milk grain stage
shoot axis at milk grain stage
glumes at milk grain stage
peduncle at milk grain stage
lemma at milk grain stage
awns at milk grain stage
grain at milk grain stage
flag leaf blade (senescence) at Dough
embryo proper at Dough
endosperm at Dough
grain at Soft dough
grain at Hard dough
flag leaf blade (senescence) at Ripening
grain at Ripening
Tissue

J.

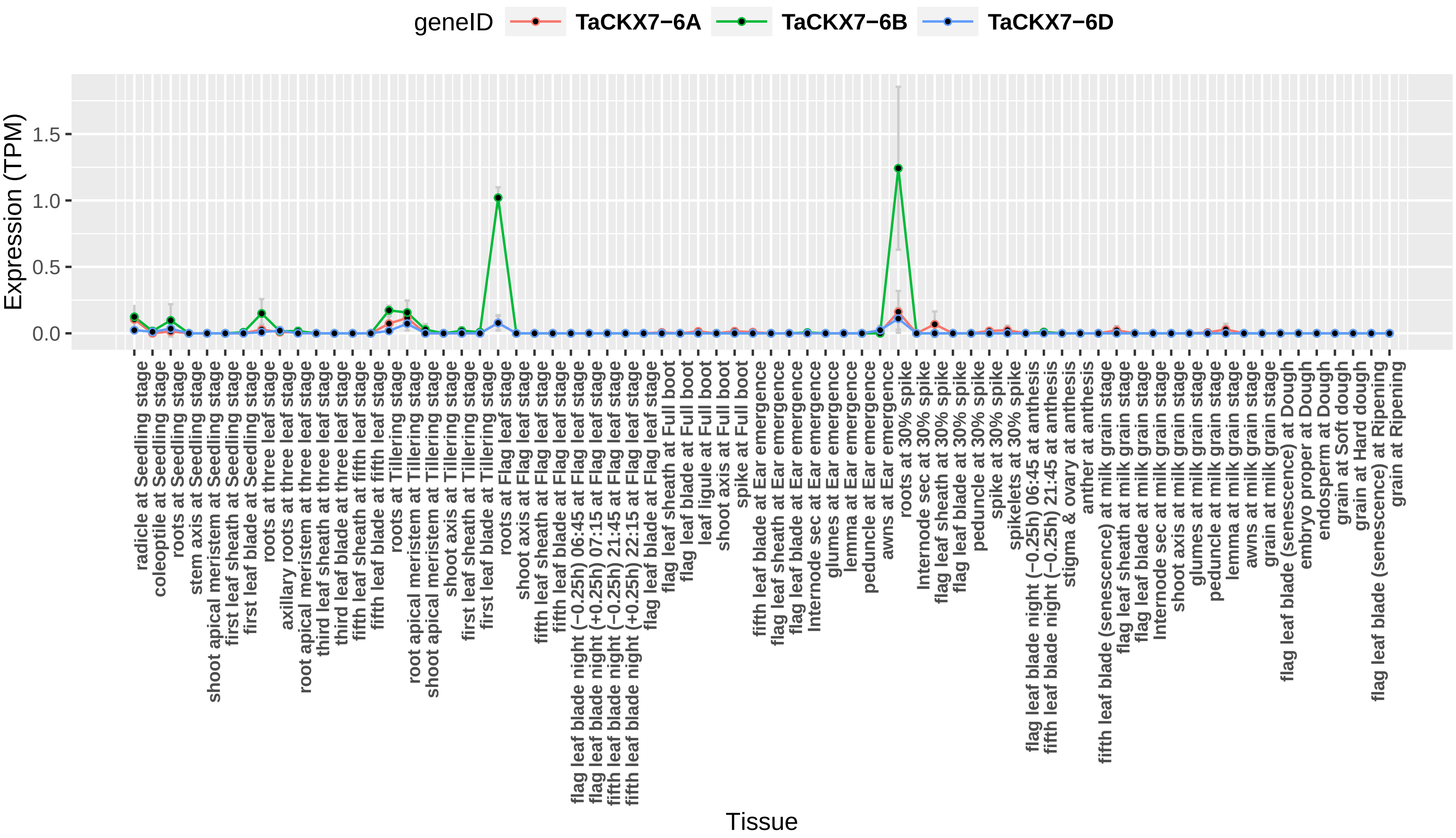

geneID
TaCKX7−6A
TaCKX7−6B
TaCKX7−6D
Expression (TPM)
0.0
0.5
1.0
1.5
Tissue
radicle at Seedling stage
coleoptile at Seedling stage
roots at Seedling stage
stem axis at Seedling stage
shoot apical meristem at Seedling stage
first leaf sheath at Seedling stage
first leaf blade at Seedling stage
roots at three leaf stage
axillary roots at three leaf stage
root apical meristem at three leaf stage
third leaf sheath at three leaf stage
third leaf blade at three leaf stage
fifth leaf sheath at fifth leaf stage
fifth leaf blade at fifth leaf stage
roots at Tillering stage
root apical meristem at Tillering stage
shoot apical meristem at Tillering stage
shoot axis at Tillering stage
first leaf sheath at Tillering stage
first leaf blade at Tillering stage
roots at Flag leaf stage
shoot axis at Flag leaf stage
fifth leaf sheath at Flag leaf stage
fifth leaf blade at Flag leaf stage
flag leaf blade night (−0.25h) 06:45 at Flag leaf stage
flag leaf blade night (+0.25h) 07:15 at Flag leaf stage
fifth leaf blade night (−0.25h) 21:45 at Flag leaf stage
fifth leaf blade night (+0.25h) 22:15 at Flag leaf stage
flag leaf blade at Flag leaf stage
flag leaf sheath at Full boot
flag leaf blade at Full boot
leaf ligule at Full boot
shoot axis at Full boot
spike at Full boot
fifth leaf blade at Ear emergence
flag leaf sheath at Ear emergence
flag leaf blade at Ear emergence
Internode sec at Ear emergence
glumes at Ear emergence
lemma at Ear emergence
peduncle at Ear emergence
awns at Ear emergence
roots at 30% spike
Internode sec at 30% spike
flag leaf sheath at 30% spike
flag leaf blade at 30% spike
peduncle at 30% spike
spike at 30% spike
spikelets at 30% spike
flag leaf blade night (−0.25h) 06:45 at anthesis
fifth leaf blade night (−0.25h) 21:45 at anthesis
stigma & ovary at anthesis
anther at anthesis
fifth leaf blade (senescence) at milk grain stage
flag leaf sheath at milk grain stage
flag leaf blade at milk grain stage
Internode sec at milk grain stage
shoot axis at milk grain stage
glumes at milk grain stage
peduncle at milk grain stage
lemma at milk grain stage
awns at milk grain stage
grain at milk grain stage
flag leaf blade (senescence) at Dough
embryo proper at Dough
endosperm at Dough
grain at Soft dough
grain at Hard dough
flag leaf blade (senescence) at Ripening
grain at Ripening

K.

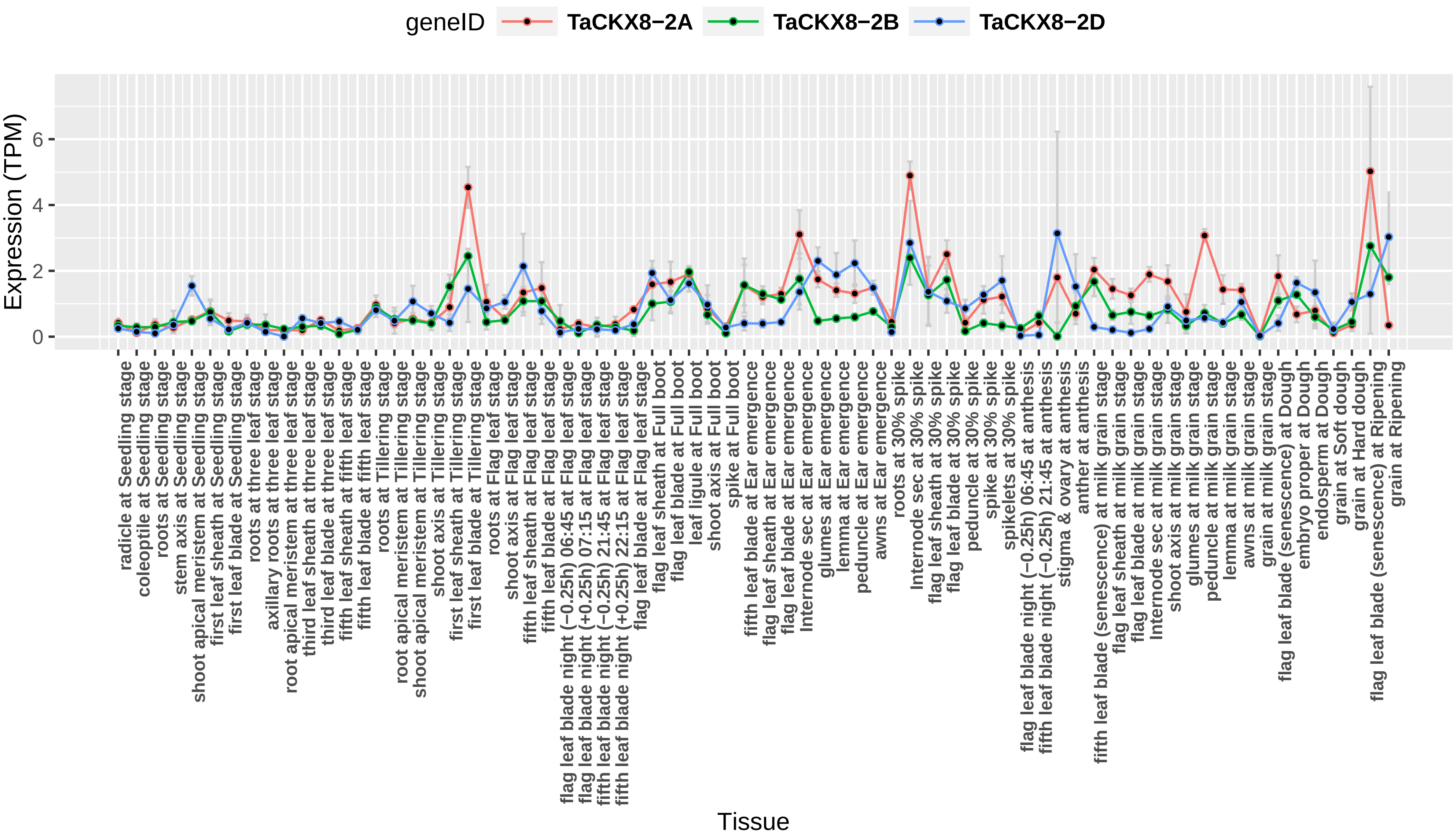
geneID
TaCKX8−2A
TaCKX8−2B
TaCKX8−2D
Expression (TPM)
0
2
4
6
radicle at Seedling stage
coleoptile at Seedling stage
roots at Seedling stage
stem axis at Seedling stage
shoot apical meristem at Seedling stage
first leaf sheath at Seedling stage
first leaf blade at Seedling stage
roots at three leaf stage
axillary roots at three leaf stage
root apical meristem at three leaf stage
third leaf sheath at three leaf stage
third leaf blade at three leaf stage
fifth leaf sheath at fifth leaf stage
fifth leaf blade at fifth leaf stage
roots at Tillering stage
root apical meristem at Tillering stage
shoot apical meristem at Tillering stage
shoot axis at Tillering stage
first leaf sheath at Tillering stage
first leaf blade at Tillering stage
roots at Flag leaf stage
shoot axis at Flag leaf stage
fifth leaf sheath at Flag leaf stage
fifth leaf blade at Flag leaf stage
flag leaf blade night (−0.25h) 06:45 at Flag leaf stage
flag leaf blade night (+0.25h) 07:15 at Flag leaf stage
fifth leaf blade night (−0.25h) 21:45 at Flag leaf stage
fifth leaf blade night (+0.25h) 22:15 at Flag leaf stage
flag leaf blade at Flag leaf stage
flag leaf sheath at Full boot
flag leaf blade at Full boot
leaf ligule at Full boot
shoot axis at Full boot
spike at Full boot
fifth leaf blade at Ear emergence
flag leaf sheath at Ear emergence
flag leaf blade at Ear emergence
Internode sec at Ear emergence
glumes at Ear emergence
lemma at Ear emergence
peduncle at Ear emergence
awns at Ear emergence
roots at 30% spike
Internode sec at 30% spike
flag leaf sheath at 30% spike
flag leaf blade at 30% spike
peduncle at 30% spike
spike at 30% spike
spikelets at 30% spike
flag leaf blade night (−0.25h) 06:45 at anthesis
fifth leaf blade night (−0.25h) 21:45 at anthesis
stigma & ovary at anthesis
anther at anthesis
fifth leaf blade (senescence) at milk grain stage
flag leaf sheath at milk grain stage
flag leaf blade at milk grain stage
Internode sec at milk grain stage
shoot axis at milk grain stage
glumes at milk grain stage
peduncle at milk grain stage
lemma at milk grain stage
awns at milk grain stage
grain at milk grain stage
flag leaf blade (senescence) at Dough
embryo proper at Dough
endosperm at Dough
grain at Soft dough
grain at Hard dough
flag leaf blade (senescence) at Ripening
grain at Ripening
Tissue

L.

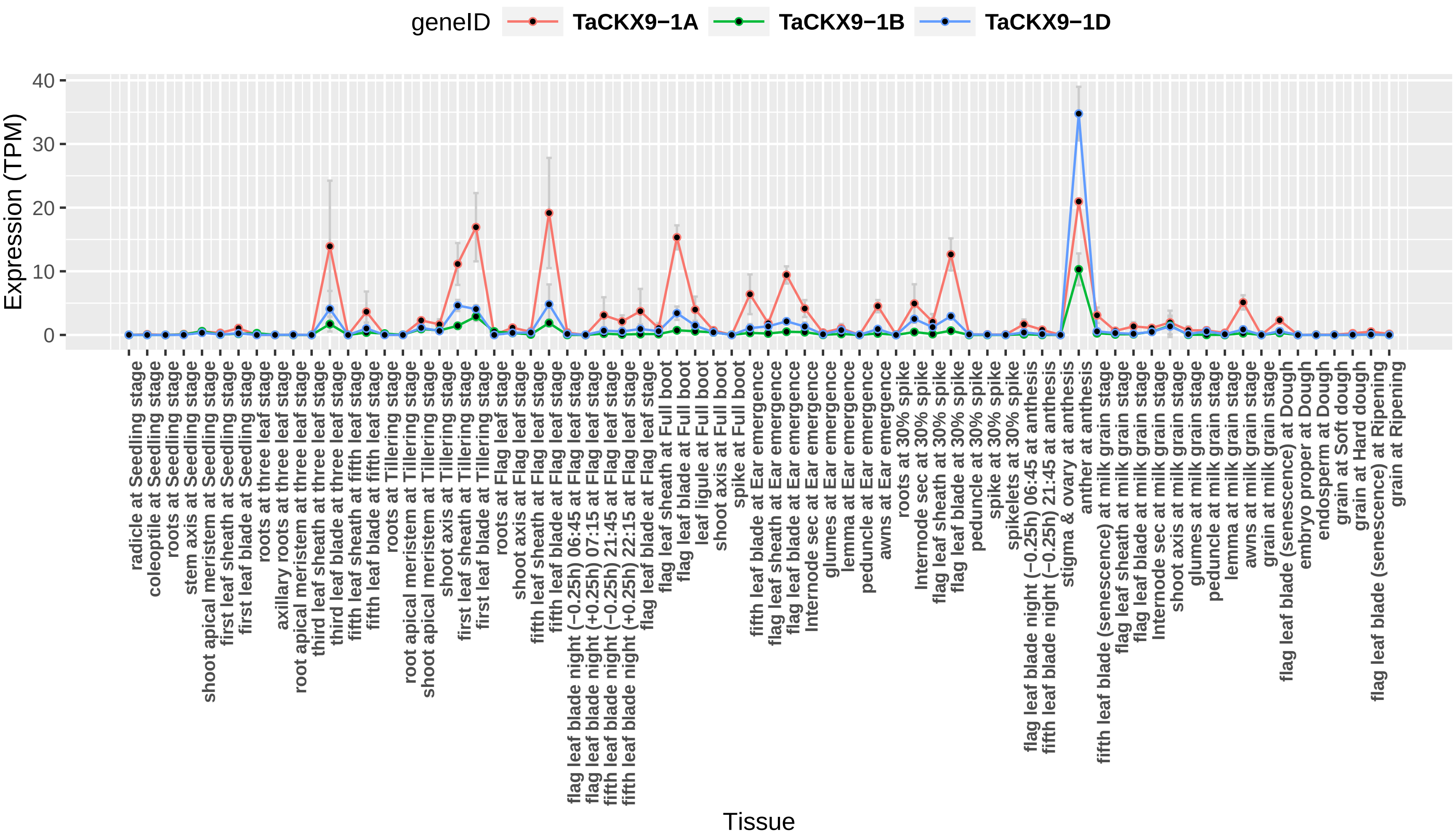

geneID
TaCKX9−1A
TaCKX9−1B
TaCKX9−1D
Expression (TPM)
40
30
20
10
0
Tissue
radicle at Seedling stage
coleoptile at Seedling stage
roots at Seedling stage
stem axis at Seedling stage
shoot apical meristem at Seedling stage
first leaf sheath at Seedling stage
first leaf blade at Seedling stage
roots at three leaf stage
axillary roots at three leaf stage
root apical meristem at three leaf stage
third leaf sheath at three leaf stage
third leaf blade at three leaf stage
fifth leaf sheath at fifth leaf stage
fifth leaf blade at fifth leaf stage
roots at Tillering stage
root apical meristem at Tillering stage
shoot apical meristem at Tillering stage
shoot axis at Tillering stage
first leaf sheath at Tillering stage
first leaf blade at Tillering stage
roots at Flag leaf stage
shoot axis at Flag leaf stage
fifth leaf sheath at Flag leaf stage
fifth leaf blade at Flag leaf stage
flag leaf blade night (−0.25h) 06:45 at Flag leaf stage
flag leaf blade night (+0.25h) 07:15 at Flag leaf stage
fifth leaf blade night (−0.25h) 21:45 at Flag leaf stage
fifth leaf blade night (+0.25h) 22:15 at Flag leaf stage
flag leaf blade at Flag leaf stage
flag leaf sheath at Full boot
flag leaf blade at Full boot
leaf ligule at Full boot
shoot axis at Full boot
spike at Full boot
fifth leaf blade at Ear emergence
flag leaf sheath at Ear emergence
flag leaf blade at Ear emergence
Internode sec at Ear emergence
glumes at Ear emergence
lemma at Ear emergence
peduncle at Ear emergence
awns at Ear emergence
roots at 30% spike
Internode sec at 30% spike
flag leaf sheath at 30% spike
flag leaf blade at 30% spike
peduncle at 30% spike
spike at 30% spike
spikelets at 30% spike
flag leaf blade night (−0.25h) 06:45 at anthesis
fifth leaf blade night (−0.25h) 21:45 at anthesis
stigma & ovary at anthesis
anther at anthesis
fifth leaf blade (senescence) at milk grain stage
flag leaf sheath at milk grain stage
flag leaf blade at milk grain stage
Internode sec at milk grain stage
shoot axis at milk grain stage
glumes at milk grain stage
peduncle at milk grain stage
lemma at milk grain stage
awns at milk grain stage
grain at milk grain stage
flag leaf blade (senescence) at Dough
embryo proper at Dough
endosperm at Dough
grain at Soft dough
grain at Hard dough
flag leaf blade (senescence) at Ripening
grain at Ripening

M.

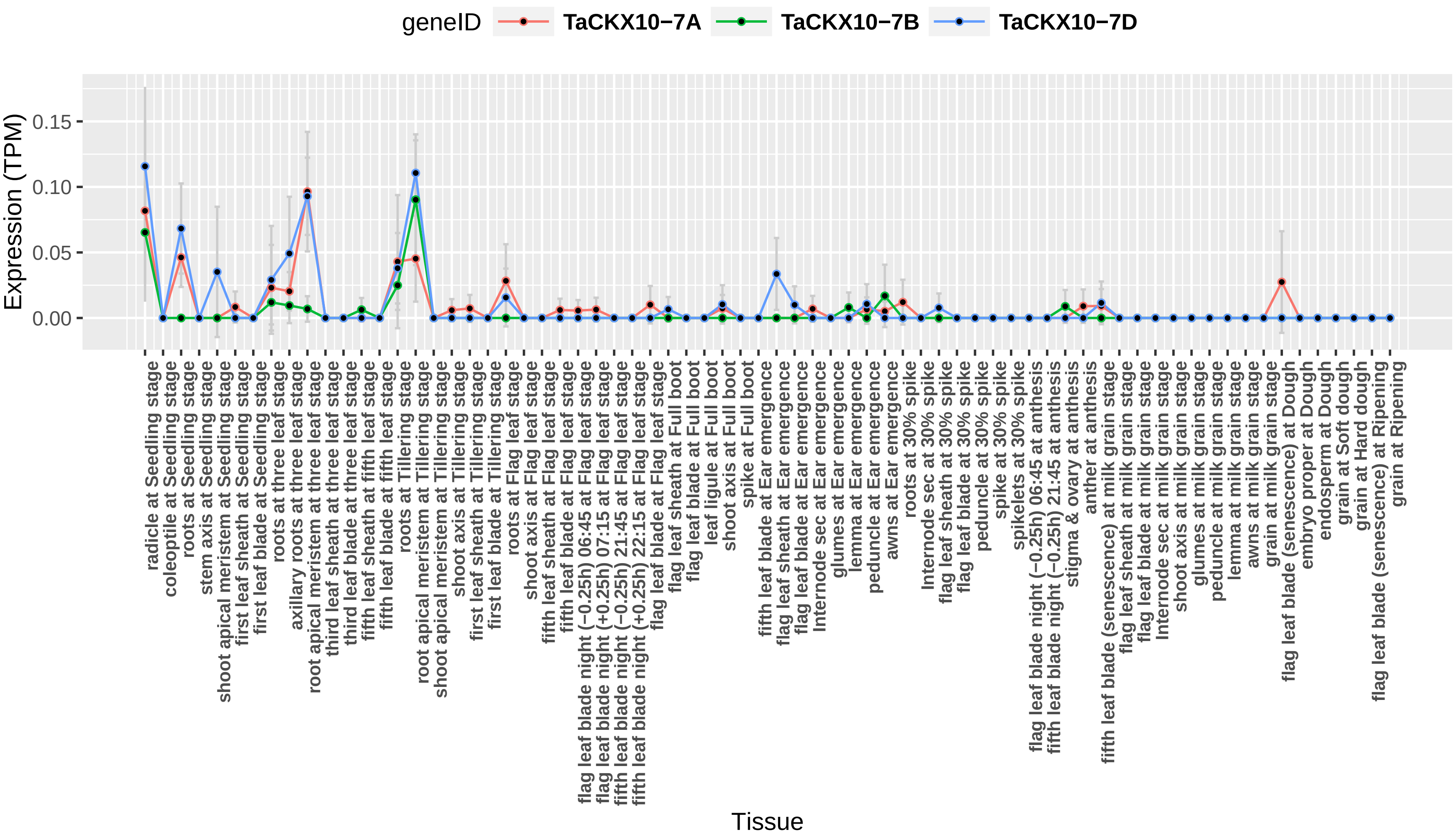
geneID
TaCKX10−7A
TaCKX10−7B
TaCKX10−7D
Expression (TPM)
0.15
0.10
0.05
0.00
Tissue
radicle at Seedling stage
coleoptile at Seedling stage
roots at Seedling stage
stem axis at Seedling stage
shoot apical meristem at Seedling stage
first leaf sheath at Seedling stage
first leaf blade at Seedling stage
roots at three leaf stage
axillary roots at three leaf stage
root apical meristem at three leaf stage
third leaf sheath at three leaf stage
third leaf blade at three leaf stage
fifth leaf sheath at fifth leaf stage
fifth leaf blade at fifth leaf stage
roots at Tillering stage
root apical meristem at Tillering stage
shoot apical meristem at Tillering stage
shoot axis at Tillering stage
first leaf sheath at Tillering stage
first leaf blade at Tillering stage
roots at Flag leaf stage
shoot axis at Flag leaf stage
fifth leaf sheath at Flag leaf stage
fifth leaf blade at Flag leaf stage
flag leaf blade night (−0.25h) 06:45 at Flag leaf stage
flag leaf blade night (+0.25h) 07:15 at Flag leaf stage
fifth leaf blade night (−0.25h) 21:45 at Flag leaf stage
fifth leaf blade night (+0.25h) 22:15 at Flag leaf stage
flag leaf blade at Flag leaf stage
flag leaf sheath at Full boot
flag leaf blade at Full boot
leaf ligule at Full boot
shoot axis at Full boot
spike at Full boot
fifth leaf blade at Ear emergence
flag leaf sheath at Ear emergence
flag leaf blade at Ear emergence
Internode sec at Ear emergence
glumes at Ear emergence
lemma at Ear emergence
peduncle at Ear emergence
awns at Ear emergence
roots at 30% spike
Internode sec at 30% spike
flag leaf sheath at 30% spike
flag leaf blade at 30% spike
peduncle at 30% spike
spike at 30% spike
spikelets at 30% spike
flag leaf blade night (−0.25h) 06:45 at anthesis
fifth leaf blade night (−0.25h) 21:45 at anthesis
stigma & ovary at anthesis
anther at anthesis
fifth leaf blade (senescence) at milk grain stage
flag leaf sheath at milk grain stage
flag leaf blade at milk grain stage
Internode sec at milk grain stage
shoot axis at milk grain stage
glumes at milk grain stage
peduncle at milk grain stage
lemma at milk grain stage
awns at milk grain stage
grain at milk grain stage
flag leaf blade (senescence) at Dough
embryo proper at Dough
endosperm at Dough
grain at Soft dough
grain at Hard dough
flag leaf blade (senescence) at Ripening
grain at Ripening

N.

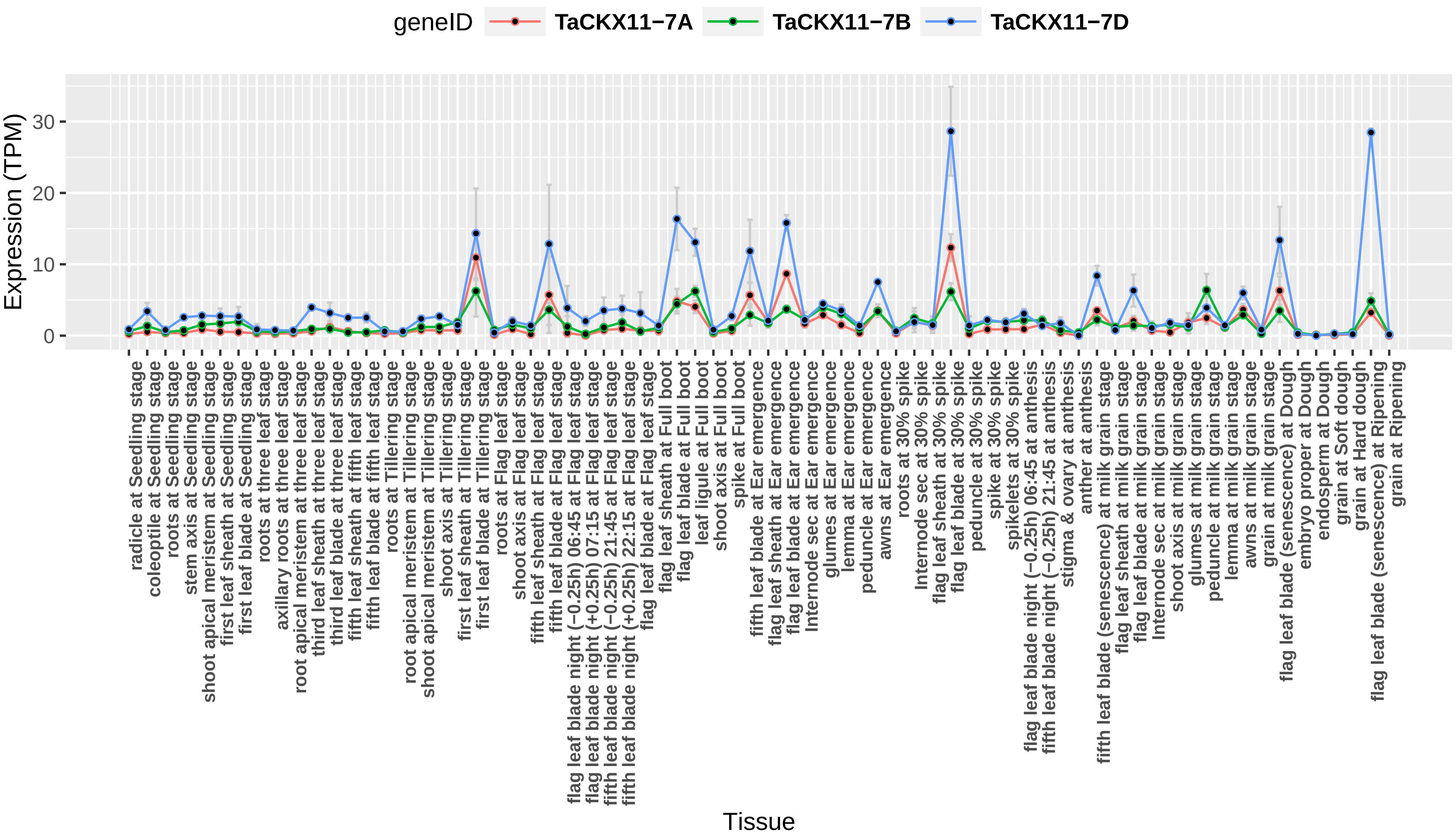

geneID
TaCKX11−7A
TaCKX11−7B
TaCKX11−7D
Expression (TPM)
0
10
20
30
Tissue
radicle at Seedling stage
coleoptile at Seedling stage
roots at Seedling stage
stem axis at Seedling stage
shoot apical meristem at Seedling stage
first leaf sheath at Seedling stage
first leaf blade at Seedling stage
roots at three leaf stage
axillary roots at three leaf stage
root apical meristem at three leaf stage
third leaf sheath at three leaf stage
third leaf blade at three leaf stage
fifth leaf sheath at fifth leaf stage
fifth leaf blade at fifth leaf stage
roots at Tillering stage
root apical meristem at Tillering stage
shoot apical meristem at Tillering stage
shoot axis at Tillering stage
first leaf sheath at Tillering stage
first leaf blade at Tillering stage
roots at Flag leaf stage
shoot axis at Flag leaf stage
fifth leaf sheath at Flag leaf stage
fifth leaf blade at Flag leaf stage
flag leaf blade night (−0.25h) 06:45 at Flag leaf stage
flag leaf blade night (+0.25h) 07:15 at Flag leaf stage
fifth leaf blade night (−0.25h) 21:45 at Flag leaf stage
fifth leaf blade night (+0.25h) 22:15 at Flag leaf stage
flag leaf blade at Flag leaf stage
flag leaf sheath at Full boot
flag leaf blade at Full boot
leaf ligule at Full boot
shoot axis at Full boot
spike at Full boot
fifth leaf blade at Ear emergence
flag leaf sheath at Ear emergence
flag leaf blade at Ear emergence
Internode sec at Ear emergence
glumes at Ear emergence
lemma at Ear emergence
peduncle at Ear emergence
awns at Ear emergence
roots at 30% spike
Internode sec at 30% spike
flag leaf sheath at 30% spike
flag leaf blade at 30% spike
peduncle at 30% spike
spike at 30% spike
spikelets at 30% spike
flag leaf blade night (−0.25h) 06:45 at anthesis
fifth leaf blade night (−0.25h) 21:45 at anthesis
stigma & ovary at anthesis
anther at anthesis
fifth leaf blade (senescence) at milk grain stage
flag leaf sheath at milk grain stage
flag leaf blade at milk grain stage
Internode sec at milk grain stage
shoot axis at milk grain stage
glumes at milk grain stage
peduncle at milk grain stage
lemma at milk grain stage
awns at milk grain stage
grain at milk grain stage
flag leaf blade (senescence) at Dough
embryo proper at Dough
endosperm at Dough
grain at Soft dough
grain at Hard dough
flag leaf blade (senescence) at Ripening
grain at Ripening

Fig S2. RNA-seq graphs for all *TaCKX* gene family members. The raw datasets were collected from IWGSC (Science 361, 2018) and calculated by Wheat Omics of China. We extracted the *TaCKX* gene expression dataset by awk, python and perl programs. The figure was drawn used the expression mean and SD values used the ggplot2 package in R language.
